# Fetal whole‐heart 4D imaging using motion‐corrected multi‐planar real‐time MRI

**DOI:** 10.1002/mrm.27798

**Published:** 2019-05-12

**Authors:** Joshua F.P. van Amerom, David F.A. Lloyd, Maria Deprez, Anthony N. Price, Shaihan J. Malik, Kuberan Pushparajah, Milou P.M. van Poppel, Mary A. Rutherford, Reza Razavi, Joseph V Hajnal

**Affiliations:** ^1^ School of Biomedical Engineering & Imaging Sciences King’s College London London United Kingdom; ^2^ Department of Congenital Heart Disease Evelina Children’s Hospital London United Kingdom; ^3^ Centre for the Developing Brain King’s College London London United Kingdom

**Keywords:** cardiac MRI, congenital heart disease, 4D reconstruction, fetal imaging, motion correction

## Abstract

**Purpose:**

To develop an MRI acquisition and reconstruction framework for volumetric cine visualization of the fetal heart and great vessels in the presence of maternal and fetal motion.

**Methods:**

Four‐dimensional (4D) depiction was achieved using a highly‐accelerated multi‐planar real‐time balanced steady‐state free precession acquisition combined with retrospective image‐domain techniques for motion correction, cardiac synchronization and outlier rejection. The framework was validated using a numerical phantom and evaluated in a study of 20 mid‐ to late‐gestational age human fetal subjects (23‐33 weeks gestational age). Reconstructed MR data were compared with matched ultrasound. A preliminary assessment of flow‐sensitive reconstruction using the velocity information encoded in the phase of real‐time images is included.

**Results:**

Reconstructed 4D data could be visualized in any two‐dimensional plane without the need for highly specific scan plane prescription prior to acquisition or for maternal breath hold to minimize motion. Reconstruction was fully automated aside from user‐specified masks of the fetal heart and chest. The framework proved robust when applied to fetal data and simulations confirmed that spatial and temporal features could be reliably recovered. Evaluation suggested the reconstructed framework has the potential to be used for comprehensive assessment of the fetal heart, either as an adjunct to ultrasound or in combination with other MRI techniques.

**Conclusions:**

The proposed methods show promise as a framework for motion‐compensated 4D assessment of the fetal heart and great vessels.

## 1 INTRODUCTION

Fetal cardiac MRI has been limited by the challenges associated with imaging a small, rapidly beating heart that is subject to various regular and spontaneous movements within the maternal torso.^1^, ^2^ However, as motion‐tolerant fetal cardiac MR methods are introduced^3^, ^4^ there is an increasing capacity for MRI to visualize the fetal heart and great vessels. The success of retrospective methods for high‐resolution 3D depiction of the fetal brain^5^, ^6^ suggests the potential of this approach for 4D reconstruction of the fetal heart in the presence of motion, allowing for robust cardiac evaluation.

Recent published studies have taken advantage of the flexibility of continuous golden angle radial sampling and the resolution that can be achieved with segmented cine imaging using compressed sensing for 2D imaging of the fetal heart,^7^, ^8^, ^9^ with impressive results. In‐plane motion‐correction in k‐space is possible with this approach,^3^ however through‐plane fetal and maternal movements cannot currently be corrected and in‐plane motion across the entire field of view must be dealt with prior to or during cine MR image reconstruction.

This work builds on the motion‐tolerant image‐domain cine reconstruction framework previously described for 2D cardiac fetal imaging^4^ and methods for 3D image volume reconstruction,^5^ with the aim of developing an acquisition and reconstruction approach to generate a 4D cine representation of the fetal cardiovascular anatomy in utero from 2D multi‐planar real‐time MR images, without the need for maternal breath‐hold or significant manual processing. Whole heart coverage presents challenges beyond 2D imaging, as motion must be corrected or compensated and the cardiac cycle synchronized for all acquired data is needed to allow for volumetric reconstruction.

In this work, 4D capability was achieved using a multi‐planar real‐time acquisition combined with retrospective image‐domain techniques for motion correction, cardiac synchronization and outlier rejection. Preliminary results showed the proposed framework was capable of reconstructing 4D data from real‐time MR images acquired without maternal breath‐hold and without requiring precise scan plane prescription during acquisition.^10^ In this work, the framework was validated using numerically simulated cardiac MRI data and evaluated in a study of 20 human fetal subjects, including comparison with matched ultrasound. A preliminary assessment of velocity‐sensitive reconstruction was also performed.

## 2 METHODS

**Figure 1:**
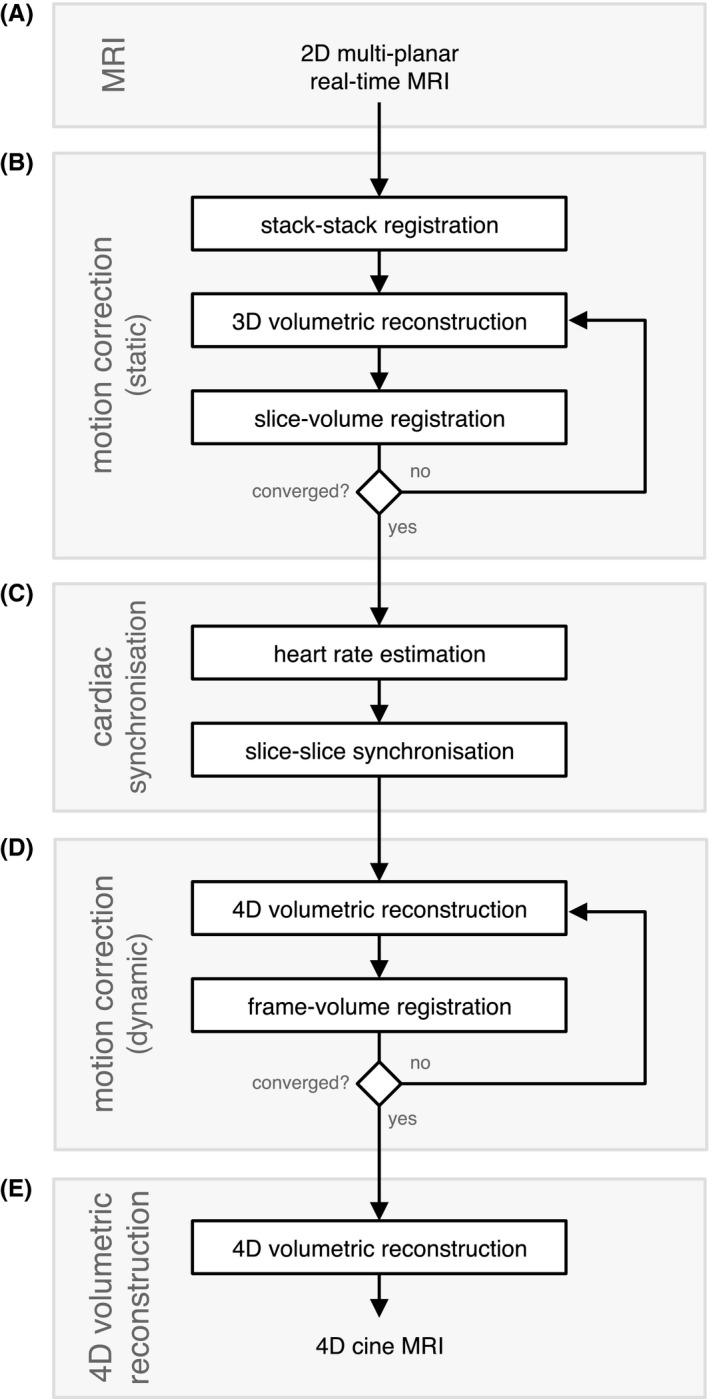
Framework for 4D cine volumetric reconstruction, consisting of (A) acquisition and reconstruction of multi‐planar real‐time 2D MRI; (B) an initial motion correction stage to achieve rough spatial alignment of the fetal heart using temporal mean (i.e., static) images for stack‐stack registration followed by slice‐volume registration interleaved with static volume (3D) reconstruction; (C) cardiac synchronization, including heart rate estimation and slice‐slice cardiac cycle alignment; and (D) further motion‐correction using real‐time (i.e., dynamic) image frames interleaved with 4D reconstruction; and (E) a final volumetric reconstruction, including outlier rejection. User‐specified ROIs, and identification of a target stack for stack‐stack registration are the only manual preparations required for reconstruction

The proposed framework for 4D whole‐heart reconstruction is shown in Figure 1 and consists of motion correction using ‘static’ temporal mean images to achieve rough spatial alignment, followed by cardiac synchronization and further motion correction using ‘dynamic’ real‐time images, and concluding with a final 4D reconstruction including outlier rejection. The symbols used to denote the acquired real‐time MR images and reconstructed 4D cine MRI throughout this manuscript follow the convention commonly used for slice‐to‐volume reconstruction (SVR) methods,^5^, ^6^ which differs from those used previously to describe the purely 2D cine framework.^4^

### 2.1 Multi‐planar real‐time MRI

Multi‐planar data were acquired in stacks of parallel slices, as in previous SVR reconstruction methods.^5^ Stacks were acquired in multiple orientations, ideally with the initial three stacks planned mutually orthogonal to each other to ensure full coverage of cardiac anatomy. For each slice a series of real‐time images (or frames) was obtained, thus ensuring dense sampling in both space and time. Fetal and maternal movement were expected to cause shifting of the fetal heart relative to the imaging field of view. These shifts can be large on the pixel scale and in any direction.

The acquired data form a set of $N_{k}$ real‐time MR image frames, $Y\;=\;{\left\{Y_{k}\right\}}_{k\;=\;1,\;\cdots ,\;N_{k}}$, where each frame has an associated acquisition time $t_{k}$ and consists of elements $y_{\mathrm{jk}}$ at 2D spatial coordinates indexed by *j*. Subsets of **Y** for a single slice are defined as $Y_{l}\;=\;{\left\{Y_{k}\right\}}_{k\;\in \;{\text{slice}}_{l}}$ where parallel $Y_{l}$ form a stack.

In the previous 2D+time framework^4^ real‐time MR images were reconstructed using k‐t SENSE^11^ with spatially‐adaptive regularization to preserve the full temporal resolution of the accelerated acquisition. These complex‐valued real‐time images were then combined as cine image series using robust statistics based on complex‐valued errors to suppress voxels corrupted by artefact. This was possible because the phase of the complex‐valued real‐time images was fairly consistent in a single slice. However, in this 4D framework, the phase of the real‐time images is inconsistent due to changes in slice orientation and position. As a result, these complex‐valued images cannot be directly combined as coherent complex‐valued volumetric data and magnitude‐valued images were used. Consequently, magnitude‐valued images from a k‐t SENSE reconstruction were used to simplify the reconstruction methods and focus on the key challenge of locating slices in 3D space and time to reconstruct 4D cine representations of the fetal cardiovascular system.

Motion correction, cardiac synchronization and volumetric reconstruction were all performed on a region of interest (ROI) centred on the fetal heart. In this work static ROIs covering the heart, great vessels and arterial arches were manually prescribed for each slice l. A fetal chest volume of interest and target stack used for motion correction were also user‐specified. These were the only manual preparations required. Fetal heart ROIs were prescribed in a few seconds each, while the fetal chest volume of interest was specified in a few minutes.

### 2.2 Volumetric reconstruction

Reconstruction of a 4D cine from 2D MR images requires information about the spatial and temporal position of the heart in each acquired 2D image frame. While the slice position and acquisition times are known, the relative spatial displacement due to fetal‐maternal motion and the cardiac phase are not. However, if the spatial and temporal positions can be accurately estimated, the spatial location of the image frames can be transformed using rigid transformation matrices $A\;=\;{\left\{A_{k}\right\}}_{k\;=\;1,\;\cdots ,\;N_{k}}$, assuming rigid‐body displacement, and acquisition times can be mapped to cardiac phases, $\theta \;=\;{\left\{\theta_{k}\right\}}_{k\;=\;1,\;\cdots ,\;N_{k}}$, with $\theta_{k}$ defined on a cyclic interval [0, 2*π*].

Image domain volumetric reconstruction is based on forward modelling of the image acquisition process, with volumetric reconstruction formulated as the solution to an inverse problem. In this work the aim was to recover a 4D representation the beating fetal heart. The MR image acquisition model previously used for static anatomy^5^ was modified to include a temporal component, assuming the periodic motion of the cardiac anatomy can be characterized by a cardiac phase so that all acquired real‐time images can be combined as a single cardiac cycle. This MR image acquisition model describes the relationship between MR image frames, $Y_{k}$, and a high‐resolution 4D cine, $X\;=\;{\left\{X_{h}\right\}}_{h\;=\;1,\;\cdots ,\;N_{h}}$, with three spatial dimensions and a fourth periodic temporal dimension,

(1) $$Y_{k}\;=\;\sum_{h}W^{\mathrm{hk}}X_{h}$$

where $X_{h}$ has elements $x_{\mathrm{ih}}$ for spatial index *i* and temporal index *h* corresponding to cardiac phases $\vartheta \;=\;{\left\{\vartheta_{h}\right\}}_{h\;=\;1,\;\cdots ,\;N_{h}}$, and matrices $W^{\mathrm{hk}}$ are the product of a spatial weight, $M^{k}$, and a temporal weight, $d^{\mathrm{hk}}$, so that $w_{\mathrm{ij}}^{\mathrm{hk}}\;=\;d^{\mathrm{hk}}m_{\mathrm{ij}}^{k}$. Each row of $M^{k}$ is made of coefficients ${\left\{m_{\mathrm{ij}}^{k}\right\}}_{i\;=\;1,\;\cdots ,\;N_{i}}$ that relate the spatial locations of 4D cine **X** and MR image frame $Y_{k}$, taking in to account spatial blurring and down‐sampling, as well as the movement defined by spatial transformation $A_{k}$. A point spread function (PSF), determined by the MR acquisition, forms the basis for $W^{\mathrm{hk}}$. The spatial in‐plane and through‐plane PSF were approximated as Gaussian,^5^, ^12^ while the temporal PSF for the band‐limited k‐t SENSE reconstruction was a sinc.^4^

An initial estimate of **X** was obtained by PSF‐weighted interpolation of the scattered data.^5^, ^13^ The error between acquired image frames and the image frames predicted by the acquisition model (Equation 1), given an estimate of **X**, was calculated as

(2) $$e_{\mathrm{jk}}\;=\;y_{\mathrm{jk}}^{*}-\sum_{j}\sum_{k}w_{\mathrm{ij}}^{\mathrm{hk}}x_{\mathrm{ij}},$$

where $y_{\mathrm{jk}}^{*}$ are the elements of acquired image frames intensity‐matched to account for variation in signal scaling and differential bias fields.^5^ Robust statistics were employed on both a voxel‐ and image frame‐wise basis to reduce the impact of data corrupted by artefact or being misplaced in space or time. The probabilities of a voxel, $p_{\mathrm{jk}}^{\mathrm{voxel}}$, or image frame, $p_{k}^{\mathrm{frame}}$, being an inlier rather than outlier were combined as $p_{\mathrm{jk}}\;=\;p_{\mathrm{jk}}^{\mathrm{voxel}}p_{k}^{\mathrm{frame}}$.

PSF‐weighted interpolation leads to blurring in the reconstructed volumetric data due to the thick slices of the acquired images. However, super‐resolution (SR) methods^5^, ^6^ can be used to recover high‐resolution spatial data. This was accomplished using gradient descent to minimize the error in Equation (2) by solving

(3) $$X\;=\;\underset{X}{\mathrm{argmin}}\left(\sum_{j}^{}\sum_{k}^{}p_{\mathrm{jk}}{∥e_{\mathrm{jk}}∥}_{2}^{2}+\lambda R\left(X\right)\right),$$

which includes a spatial edge‐preserving regularization term, *R*(**X**), to stabilize the reconstruction and a regularization controlling parameter, λ. Intensity matching, bias correction, robust statistics and **X** were updated at each iteration.^5^

### 2.3 Motion correction

To achieve the spatial coherence required for volumetric reconstruction, rigid body image registration was used to estimate transformations **A** that align real‐time image frames **Y** with 4D cine **X**. Registration was performed in three stages to facilitate convergence, as depicted in Figure 1. The temporal mean of all real‐time images in a slice, ${\bar{Y}}_{l}$, was used as a static reference free from cardiac pulsation. Use of static images for the initial stages of motion correction allowed for gross spatial alignment, facilitating cardiac synchronization between slices and providing an initial estimate of **A** prior to motion correction of individual image frames.

Stacks were first aligned by stack‐stack registration with a target stack as in other volumetric reconstruction work,^5^, ^12^, ^13^ using static temporal mean images $\bar{Y}$.

Using the transformations estimated by stack‐stack registration, an initial static volume was reconstructed from $\bar{Y}$ and then each slice $Y_{l}$ was registered to the volume. Interleaved volume reconstruction and slice‐volume registration was repeated over multiple iterations to establish slice‐wise alignment.

Frame‐wise spatial alignment was performed following cardiac synchronization. An initial 4D cine was reconstructed using estimated cardiac phases, ***θ***, and slice‐wise transformations, **A**. Subsequently, frame‐volume registration was performed between each real‐time image frame, $Y_{k}$ and the 4D cine frame, $X_{h}$, with matched cardiac phase, i.e., $\theta_{h}\;=\;\vartheta_{k}$, followed by volumetric reconstruction using the estimated frame‐wise transformations. Interleaved 4D reconstruction and frame‐volume registration was repeated over multiple iterations.

Displacement was used to assess the change in position of the voxels in **Y** subject to estimated transformations **A**. Global displacement, disp(**A**), was calculated as

(4) $$\text{disp}\left(A\right)\;=\;\frac{\sum_{k}\sum_{j}^{}\text{dist}\left(y_{\mathrm{jk}},A_{k}\left(y_{\mathrm{jk}}\right)\right)}{\sum_{k}N_{j}},$$

where $\text{dist}\left(y_{\mathrm{jk}},\;A_{k}\left(y_{\mathrm{jk}}\right)\right)$ is the spatial distance between the original position of voxel $y_{\mathrm{jk}}$ and the position of $y_{\mathrm{jk}}$ transformed by $A_{k}$. Similarly, deviation from the average slice transformation was used to assess the dispersion of estimated transformations. Global deviation, dev(**A**), was calculated as

(5) $$\text{dev}\left(A\right)\;=\;\frac{\sum_{k}\sum_{j}\text{dist}\left(A_{l}\left(y_{\mathrm{jk}}\right),\;A_{k}\left(y_{\mathrm{jk}}\right)\right)}{\sum_{k}N_{j}},$$

where the average slice transformation, $A_{l}$, is the $p^{\mathrm{frame}}$‐weighted Frechét mean.^14^ Slice‐wise deviation, $\text{dev}(A_{l})$, was calculated from Equation (5) by restricting the summations to image frames *k* acquired at slice location *l*.

### 2.4 Cardiac synchronization

**Figure 2:**
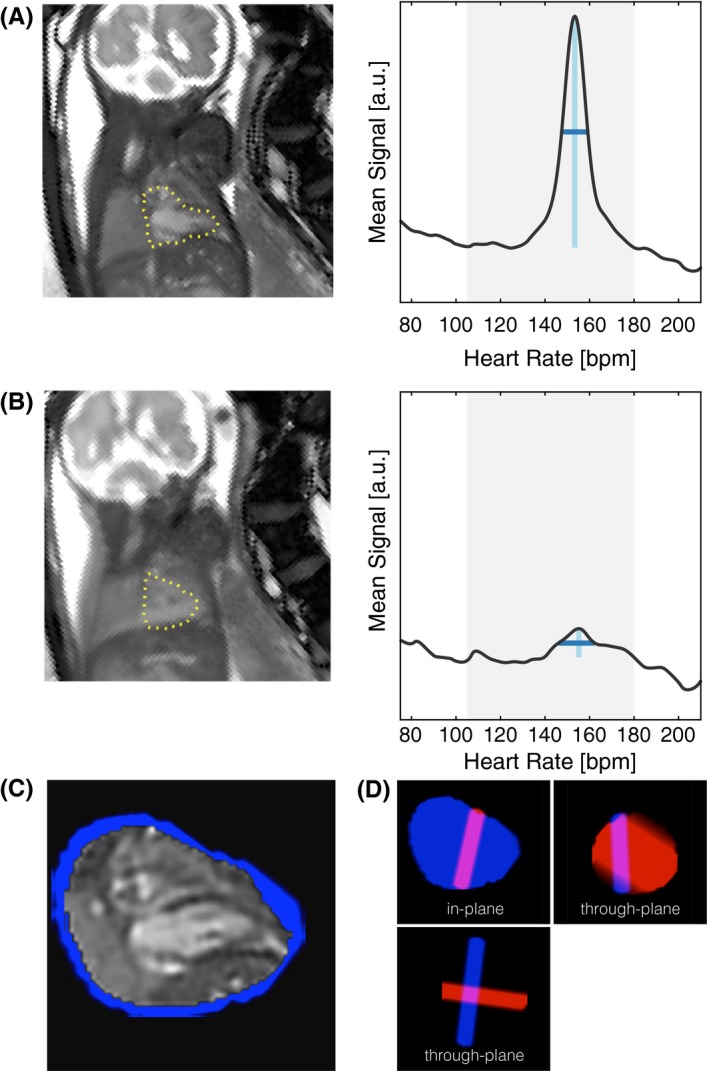
Cardiac synchronization in healthy $30^{+3}$ week gestational age fetus. Heart rate estimation: (A) Temporal mean image, ${\bar{Y}}_{l}$, for a slice with little motion ($\text{dev}\;(A_{l})\;=\;1.5$ mm) with ROI over the fetal heart (dotted yellow line), rotated to show the fetus in radiographic orientation, and plot of temporal frequency spectrum (spatial mean in the fetal heart ROI of the temporal Fourier transform of the real‐time image series) showing the clear maxima in the range of expected heart rates (grey band, 115‐180 bpm). Peak height (light blue vertical line) and width (dark blue horizontal line) are shown. (B) Fetal movement during the acquisition of a different slice from the same stack ($\text{dev}(A_{l})\;=\;9.4$ mm) leads to blurring in the temporal mean image, as well as a lower and broader peak in the temporal frequency spectrum. Unreliable heart rates, such as (B), were interpolated from temporally adjacent slices in the same stack. Slice‐slice synchronization: (C) Single frame of the cine, X_*l*_, corresponding to slice shown in (A) and (D) volume weights, V_*l*_, (blue region), as in‐plane and orthogonal through‐plane views showing intersection with a slice from a different stack (red region). Slice‐slice cardiac synchronization was performed by temporally aligning the cardiac cycles between X_*l*_ constructed from **Y**_*l*_ while using volume weights, V*l*, to determine the overlap between slices

The cardiac phase of each image frame must be known for the data to be combined as a single cardiac cycle. The heart rate was first estimated independently for each slice and then the cardiac cycle was synchronized between slices, as shown in Figure 2.

A constant heart rate was estimated for each slice from the temporal frequency spectra, i.e., the temporal Fourier transform of the real‐time image series, by identifying the maxima in the spatial mean of the temporal frequency spectrum in the fetal heart ROI^4^ (Figure 2A). These maxima were not highly sensitive to the ROI definition, however displacement of the heart and variation of the fetal heart rate resulted in diminished peaks (Figure 2B). Unreliable heart rate estimates were identified as those with peak height or width more than three normal standard deviations from the median, estimated from the median absolute deviation.^15^ If a peak could not be identified in the mean temporal frequency spectrum, the heart rate for that slice was instead calculated as the linear interpolation of reliable heart rate estimates for the slices acquired before and after that slice.

The cardiac cycle was synchronized across slices by estimating a temporal offset for each slice that aligns the pulsation of the heart in overlapping slices. To determine the overlap between slices in 3D space, real‐time MR images were cast as cines in volume space. Specifically, a 4D cine, $X_{l}$, was reconstructed for each slice, l, from the real‐time images in that slice, $Y_{l}$. Volume weights, $V_{l}$, representing the weighted contribution of the image frames acquired at slice location l in volume space, $v_{\mathrm{ihl}}\;=\;\sum_{j}\sum_{k\;\in \;{\text{slice}}_{l}}w_{\mathrm{ij}}^{\mathrm{hk}}$, were used to determine the spatial intersection of slices. The overlap between slice *l* and slice $l^{\prime }$ (Figure 2D) was calculated as $\sum_{i}\sum_{h}v_{\mathrm{ihl}}v_{ihl^{\prime }}$.

Application of a temporal offset, $\theta_{l}^{\mathrm{offset}}$, to $X_{l}$ was achieved by applying a cyclic Fourier time shift, $\varphi_{\theta^{\mathrm{offset}}}$, i.e., addition of a linear phase in the temporal frequency domain. Aligning the cardiac cycles of all the slices was equivalent to determining ${\left\{\theta_{l}^{\mathrm{offset}}\right\}}_{l\;=\;1,\;\cdots ,\;N_{l}}$ that maximized the overlap‐weighted similarity between all $\varphi_{\theta_{l}^{\mathrm{offset}}}\left(X_{l}\right)$. A group‐wise optimization was computationally intensive in practice, so a bootstrap approach was adopted instead, where one temporal offset was estimated at each iteration, i.e.,

(6) $$\underset{\theta_{l}^{\mathrm{offset}}}{\mathrm{argmin}}\;-\sum_{l^{\prime }\;\in \;\left\{l^{\prime \prime }\right\}}\frac{\sum_{i}\sum_{h}v_{\mathrm{ihl}}v_{ihl^{\prime }}\;\rho \left(\varphi_{\theta_{l}^{\mathrm{offset}}}\left(X_{l}\right),\;\varphi_{\theta_{l^{\prime }}^{\mathrm{offset}}}\left(X_{l^{\prime }}\right)\right)}{\sum_{i}\sum_{h}v_{\mathrm{ihl}}v_{ihl^{\prime }}},$$

where similarity measure *ρ* is Pearson’s correlation and $\left\{l^{\prime \prime }\right\}$ is a set of target slices. The slice $l^{\prime }$ with the greatest overlap with all other slices was assigned $\theta_{l^{\prime }}^{\mathrm{offset}}\;=\;0$, and used as an initial target slice. A different slice with the greatest overlap with *l*′ was then identified and $\theta_{l}^{\mathrm{offset}}$ was estimated by Equation (6) and cardiac phases were adjusted as $\theta_{k}\;=\;\left(\theta_{k}\;+\;\theta_{l}^{\mathrm{offset}}\right)\;mod\;2\pi$. This slice was then added to the set of fixed target slices $\left\{l^{\prime \prime }\right\}$ and the process was repeated using the next slice with the greatest overlap with $\left\{l^{\prime \prime }\right\}$. This continued until all $\theta_{l}^{\mathrm{offset}}$ were estimated.

### 2.5 Fetal study

Multi‐planar real‐time MR imaging was acquired in a cohort of 11 consecutive singleton pregnancies as part of a larger research project when examination time permitted acquisition of a minimum of three stacks with a combined total of at least 30 slices to ensure good coverage of cardiovascular anatomy. The cases in this initial cohort were used for method development and evaluation. Subsequently, data were acquired in a second cohort of 9 fetuses, 8 singleton and 1 twin pregnancy (ID13), at the discretion of the attending cardiologist without a physicist present at the examination to facilitate robust stack prescription. These cases were used to assess the utility of the methods. Fetal subjects ranged from 23 to 33 weeks gestational age. The study was conducted with the approval of the local research ethics committee and all participants gave written informed consent prior to enrolment. Details of all fetal cases are listed in Table 1, comprising 3 healthy cases and 17 with cardiac anomalies.

#### 2.5.1 Imaging

Stacks of multi‐planar real‐time images were acquired on a 1.5 T Ingenia MR system (Philips, Netherlands) using a 2D balanced steady‐state free precession (bSSFP) sequence. Highly‐precise scan plane prescription was not required, as specific views could be later obtained from the reconstructed 4D data. However, to achieve a good distribution of scan plane orientations, the first three stacks were prescribed roughly transverse, sagittal and coronal to the fetal trunk for all cases in cohort 1, with additional stacks prescribed in scanner transverse, sagittal, or coronal planes. In‐plane rotation was used to reduce the size of the field of view in the phase encode direction while avoiding alignment of pulsatile maternal anatomy (e.g., maternal descending aorta) with the fetal heart in the phase encode direction to avoid complications in the k‐t SENSE reconstruction.

**Table 1:** Fetal study subjects

ID	GA	Reason for scan	No. stacks	No. slices	US
Cohort 1					
01	$32^{+1}$	dilated aortic root	5	37	*
02	$30^{+3}$	volunteer	5	37	*
03	$24^{+6}$	volunteer	5	45	*
04	$29^{+6}$	right aortic arch	6	54	
05	$31^{+0}$	ventricular diverticulum	6	55	
06	$32^{+3}$	right aortic arch	6	75	
07	$31^{+4}$	double aortic arch	4	44	*
08	$32^{+2}$	right aortic arch	4	42	*
09	$28^{+0}$	volunteer	5	51	*
10	$31^{+4}$	double aortic arch	3	35	*
11	$33^{+2}$	cardiac tumour	4	42	
Cohort 2					
12	$32^{+0}$	interrupted aortic arch	3	25	
13	$30^{+5}$	cardiac tumour	3	39	
14	$31^{+3}$	common arterial trunk	3	33	
15	$30^{+4}$	transposition of the great arteries	4	45	
16	$31^{+6}$	right aortic arch	4	23	
17	$31^{+4}$	dextrocardia	3	34	
18	$23^{+5}$	anomalous pulmonary venous return	3	33	
19	$32^{+1}$	dilated ascending aorta	3	33	
20	$32^{+4}$	right aortic arch	3	33	

*Note*:** ID**, fetal case number; **GA**, gestational age in ${\mathrm{weeks}}^{+\mathrm{days}}$; **No. Stacks**, number of acquired multi‐planar real‐time MRI stacks; **No. Slices**, number of slices acquired across all stacks; **US**, *matched 2D and STIC ultrasound data acquired within 3 days of MRI examination; **Cohort 1**, 11 consecutive singletons scanned for method development and evaluation; **Cohort 2**, 8 singletons and 1 twin scanned for clinical assessment.

Sequence parameters were selected to balance competing goals of good signal and high spatio‐temporal resolution with full coverage of the fetomaternal anatomy in the field of view, minimal scan time and safety constraints. Scanner operation was constrained to ensure fetal and maternal safety with respect to specific absorption rate (whole body SAR <2.0 W/kg^16^), peripheral nerve stimulation (low PNS mode), and sound pressure level (SPL <85 dB(A), accounting for >30 dB attenuation in utero^17^). While necessary, safety constraints placed a limit on scanner performance and, consequently, achievable resolution and image quality.

Multi‐planar bSSFP data were collected with regular Cartesian k‐t undersampling^18^ using the following default parameters: TR/TE 3.8/1.9 ms, flip angle $60^{\circ }$, FOV 400 × 304 mm, voxel size 2.0 × 2.0 × 6.0 mm, 8 × acceleration, 72 ms temporal resolution, 96 images per slice, slice overlap 2‐3 mm. A steady state was established using an *α*/2‐TR/2 preparation pulse^19^ and dummy excitations. Coil calibration data were acquired in a scan immediately preceding the multi‐planar acquisition, and low spatial‐resolution training data was acquired immediately following the under‐sampled data. Acquisition of a typical stack took 155 s. In some cases the default FOV was either decreased or increased in the phase encode direction to accommodate maternal anatomy, with a proportional change in the temporal resolution (cohort 1 median decrease 7.6 ms, median increase 3.8 ms).

Matched ultrasound (US) was acquired on the same day or three days prior to the MRI examination for 7 cases for fetuses in cohort 1 that were part of a multimodality research protocol, as indicated in Table 1. US was performed using an EPIQ V7G system with C9‐2 curved array, V6‐2 curved volume and X6‐1 matrix array transducers (Philips, Netherlands). The echocardiography protocol included comprehensive 2D M‐mode imaging as well as B‐mode sweeps covering the fetal trunk. B‐mode images were combined using the Spatio‐Temporal Image Correlation (STIC) method, which uses image‐based estimates of the fetal heart rate to produce 4D data.^20^

#### 2.5.2 Reconstruction

All k‐t SENSE reconstructions were performed in MATLAB (Mathworks, USA), with additional functionality from ReconFrame 3.0.535 (GyroTools, Switzerland) to place the data in a common 3D space. Slice‐slice synchronization (Equation 6) was achieved by constrained non‐linear multivariate minimization using the interior point approach implemented in MATLAB’s Optimization Toolbox.

Volume reconstruction was performed using the Image Registration Toolkit (BioMedIA, UK), building on the framework implemented by Kuklisova et al^5^ with additional functionality for dynamic data. Reconstruction parameters were based on those previously described,^5^ and validated for this work using simulation experiments. Volumetric reconstruction was performed at a spatial resolution of 1.25 mm, or 5/8 of the actual acquired in‐plane resolution, similar to the ratio used for reconstruction of fetal brain MRI,^5^ and a temporal resolution corresponding to $N_{h}\;=\;\text{25}$ cardiac phases, to provide frame‐volume registration targets across the entire cardiac cycle. However, the nominal resolution of the reconstructed data was 2 × 2 × 2 mm and 72 ms. A user‐specified volume of interest covering the fetal chest was used for slice‐volume registration, making use of the anatomy surrounding the heart to facilitate the registration process, while user‐specified 2D fetal heart ROIs were combined as a volume and used for frame‐volume registration. Reconstruction code can be found at github.com/mriphysics/fetal_cmr_4d (SHA1:b6c94f073c).

#### 2.5.3 Evaluation

Whole‐heart 4D cine data were assessed to establish the quality of the reconstruction method. Three observers (DL, KP, MvP) with 4, 7, and 1 years experience reading cardiac MRI, respectively, were asked to independently navigate the reconstructed 4D cine MR data using the Medical Image Interaction Toolkit Workbench (DKFZ, Germany) and score them in 11 categories, based on the segmental approach to defining cardiac anatomy and pathology.^2^, ^21^, ^22^ Data were presented to the readers without context, though the readers may have been present at the time of scanning and may have recalled unique cases. A five‐point scale was used for scoring, as follows:

4: high‐image quality and distinct appearance of cardiac structures;
3: adequate image quality to determine most details;
2: sufficient image quality to determine some details;
1: poor image quality with significant lack of detail; and
0: inadequate image quality to visualize any cardiac structure.

Ultrasound images acquired during the broader research project were used for comparison with 4D cine data in cases where matched data were available. Left and right ventricular length and width were measured at end‐diastole and end‐systole in 4‐chamber view in the US and MR data by two readers (DL, KP) using the Medical Image Interaction Toolkit Workbench (DKFZ, Germany). Smaller features, such as vessel diameter or septal thicknesses, were not measured as they were depicted by only a few voxels in the real‐time MR images. Readers performed measurements on the same 2D M‐mode US clip, but were free to select the frames for end‐diastolic and end‐systolic measurements based on ventricular dilation and contraction. For the 4D data, the plane of the 4‐chamber view and frames were determined independently by each reader. In the MRI data, length was measured using the valve annulus as the basal point and width was measured half‐way along the length of the ventricle. The MR data was presented to the readers without context and in random order. Measurements were compared using Bland‐Altman analysis, paired two‐tailed Student’s t‐test and linear regression to evaluate inter‐observer variability and agreement between modalities.

### 2.6 Simulation

**Figure 3:**
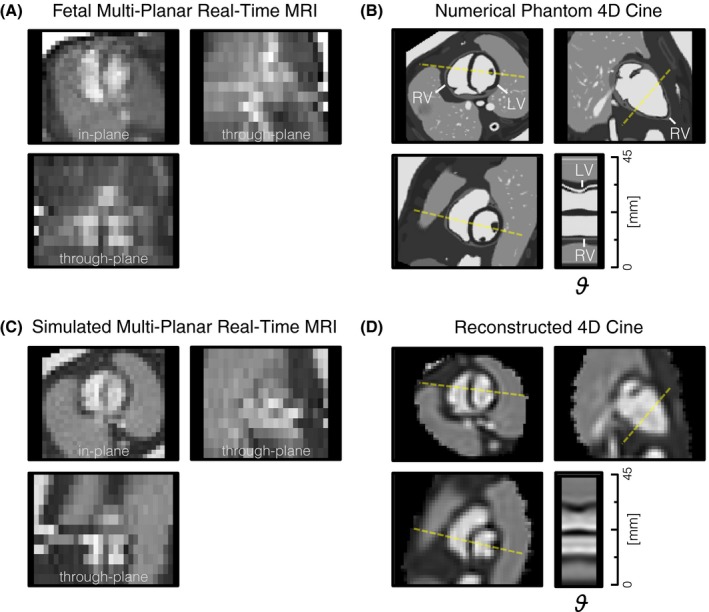
Data used to optimize and evaluate the proposed methods. (A) Real‐time MR image frame of the heart in an early third trimester fetus, acquired during the fetal study, showing misalignment of slices due to fetal movement in the through‐plane views (bottom and right). (B) High spatio‐temporal resolution 4D cine generated from numerical phantom of beating fetal heart, shown in orthogonal planes as in (A), with line profile (yellow dashed line) showing contraction and dilation of left (LV) and right (RV) ventricles across the phases (ϑ) of the cardiac cycle. (C) Simulated multi‐planar real‐time MR images derived from numerical phantom in (B) with resolution, receiver noise and motion similar to the in utero MRI in (A). (D) 4D cine reconstructed from simulated MRI data using the known cardiac phase and spatial transformation of each real‐time MR image frame

A numerically simulated phantom was used to optimize and evaluate the proposed methods under controlled conditions, as demonstrated in Figure 3. The MRXCAT phantom^23^ was used to generate a 4D cine scaled to the size of the fetal heart, ***χ***, with high spatio‐temporal resolution (0.44 mm isotropic, 25 cardiac phases) and tissue properties adapted to the in utero environment, i.e., the signal of air in the lungs, trachea and the space surrounding the body in the postnatal simulation were replaced with the signal characteristics of amniotic fluid.

Real‐time MR images with 0.5 mm in‐plane resolution and 6 mm through‐plane resolution were generated from ***χ*** using Equation (1) and further down‐sampled to 2 mm in‐plane resolution by truncating k‐space while adding noise. Cardiac phases and displacements were based on in utero measurements of the fetal heart made during the fetal study. Transformation matrices were scaled to change the amount of displacement by a scalar factor^24^ to assess varying degrees of motion. Volumetric cine data were reconstructed from simulated real‐time MR images and the results were compared with the ground truth data to evaluate the 4D reconstruction framework.

### 2.7 Velocity‐sensitive volume reconstruction

Volume reconstruction from complex‐valued real‐time images is not a straightforward extension of the proposed framework, but has the potential to generate 4D cine velocity data, i.e., 4D velocity maps, from the same images. Phase contrast bSSFP methods have been proposed,^25^ using the inherent velocity‐sensitivity of the bSSFP sequence to encode velocity in the phase images.^26^ For complex‐valued image frames, $\tilde{Y}$, the phase of an acquired voxel, $∠{\tilde{y}}_{\mathrm{jk}}$, is the sum of a velocity‐encoded phase, $\phi_{\mathrm{jk}}$, and background phase. The smoothly varying background phase can be estimated by fitting a low‐order polynomial to the phase in static tissues and subtracted from $∠{\tilde{y}}_{\mathrm{jk}}$ so that only $\phi_{\mathrm{jk}}$ remains.^27^ The $\phi_{\mathrm{jk}}$ can then be related to the underlying velocity as

(7) $$\phi_{\mathrm{jk}}\;=\;\gamma \left(\upsilon_{\mathrm{jk}}^{\mathrm{read}}M_{1}^{\mathrm{read}}+\upsilon_{\mathrm{jk}}^{\mathrm{phase}}M_{1}^{\mathrm{phase}}+\upsilon_{\mathrm{jk}}^{\mathrm{slice}}M_{1}^{\mathrm{slice}}\right)$$

where γ is the gyromagnetic ratio, and *υ* are the velocities and $M_{1}$ are the first moments of the gradient waveforms in the readout, phase‐encode and slice directions.^25^

To reconstruct volumetric velocity maps, the velocity‐encoded phase would need to be related to three‐dimensional space, and the image acquisition model (Equation 1) would have to be adjusted to include velocity terms, precipitating a number of changes to the framework. Instead, as a proof of principle, the framework was modified to reconstruct complex‐valued $\tilde{X}$. Background phase was removed from $\tilde{Y}$ by subtracting a third‐order 3D polynomial fit to the phase in static amniotic fluid and tissue in each acquired stack. A phase sign correction was applied so that the signs of all $\phi_{\mathrm{jk}}$ were aligned with the target stack, to account for differences in the direction of velocity encoding. All steps in the framework were performed as described previously, with the modification that real‐ and imaginary‐valued 4D cines were also generated separately from the real and imaginary components of $\tilde{Y}$.

## 3 RESULTS

Simulation experiments were used to evaluate the proposed method and select appropriate reconstruction parameters (Supporting Information Figures S1 and S2). The volume reconstructed from simulated MR images using the established parameters (Figure 3D) showed cardiac anatomy and pulsation matching the numerically simulated 4D cine, providing evidence of geometric accuracy. However, there was some blurring in all reconstructed 4D cines compared to the high‐resolution numerical phantom (Figure 3B), due to the low spatio‐temporal resolution sampling of the real‐time images, as well as signal inhomogeneity within the blood pool in the heart.

Fetal results are summarized in Table 2, including reader scores as an indication of image quality. 4D cines were successfully reconstructed in all 11 fetal cases in the first cohort.

**Table 2:** Summary of fetal results

ID	Reader score [0‐4]	Heart rate [bpm]	Transformations [mm]		Outliers [% *p* < 0.5]		No. Frames
			disp(**A**)	dev(**A**)	$p^{\mathrm{voxel}}$	$p^{\mathrm{frame}}$	
Cohort 1							
01	3.5	147 ± 5	4.6	1.1	0.4	5.7	3552
02	3.6	155 ± 7	5.2	1.7	0.5	9.4	3552
03	3.4	147 ± 6	7.7	1.3	1.1	7.4	3936
04	3.1	143 ± 7	11.2	1.8	0.4	21.6	3163
05	3.4	148 ± 11	12.2	2.3	0.4	10.4	5280
06	3.6	139 ± 4	7.6	1.3	0.5	15.6	6719
07	3.1	153 ± 8	8.4	2.3	0.5	28.3	3072
08	3.5	151 ± 5	4.9	1.2	0.4	20.2	3525
09	3.4	150 ± 5	2.8	1.0	1.0	7.7	4887
10	3.6	150 ± 3	4.7	1.3	0.8	4.6	3072
11	3.2	153 ± 7	5.8	2.0	1.5	43.7	4032
Median	3.4	150 ± 6	5.8	1.3	0.5	10.4	3552
Cohort 2							
12	3.2	145 ± 19	7.8	3.0	0.3	27.1	2304
13	3.6	125 ± 1	4.7	0.7	0.7	13.0	3743
14	3.4	148 ± 7	9.0	1.7	0.6	18.4	2783
15	3.6	136 ± 3	4.8	0.9	0.7	15.3	4121
16	2.5	136 ± 3	11.8	2.6	1.3	10.9	1920
17	3.9	127 ± 4	6.0	1.0	0.5	17.3	3264
18	2.1	142 ± 17	15.5	2.2	0.8	22.0	2590
19	3.9	141 ± 4	5.4	0.5	2.0	12.1	3168
20	3.8	137 ± 2	3.8	0.9	0.9	11.5	3168

*Note*:** ID**, fetal case number; **Reader Score**, mean of scores assigned across all categories by all reviewers (Cohort 1) or reviewer 1 only (Cohort 2); **Heart Rate**, mean and standard deviation of estimated fetal heart rates; **Transformations**, global displacement, disp(**A**), and deviation from average slice transformation, dev(**A**); **Outliers**, percent of voxel, $p^{\text{voxel}}$, and image frame, $p^{\text{frame}}$, probabilities below 0.5; **No. Frames**, number of image frames contributing to volume reconstruction, prior to outlier rejection. Images at the edges of the volume, i.e., those contributing fewer than $\mathrm{median}(N_{j})/10$ voxels, were excluded from these summary statistics as they had minimal impact on the reconstructed volume but were more likely to be misaligned in space or time; **Cohort 1**, consecutive singletons scanned for method development and evaluation; **Cohort 2**, fetuses scanned for clinical assessment; **Median**, Cohort 1 median values.

**Figure 4:**
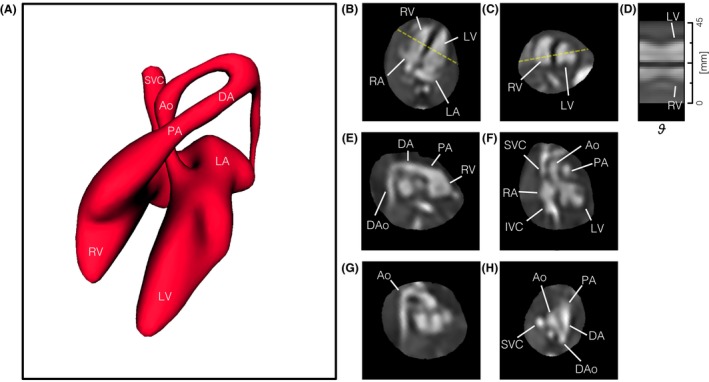
Reconstructed 4D cine of the heart of a healthy $28^{+0}$ week gestational age fetus (ID09). (A) Volume surface rendering of blood pool in diastole showing arrangement and connections of chambers and vessels, for reference. The reconstructed 4D data are shown re‐sliced in (B) 4‐chamber, (C) mid‐short axis, (E) right ventricular outflow tract, (F) left ventricular outflow tract, (G) aortic arch, and (H) 3‐vessel views. (D) A line profile at the intersection of the 4‐chamber and mid‐short axis views (dashed yellow lines) shows the contraction and dilation of the ventricles with cardiac phase (ϑ). Ventriculo‐arterial connections can be seen in outflow tract views with the pulmonary artery (PA) from the right ventricle (RV) in (E) and the aorta (Ao) arising from the left ventricle (LV) in (F). Systemic venous connections of the superior (SVC) and inferior (IVC) vena cava with the right atrium (RA) can also be seen in (F). The ductal arch (DA) can be seen in both (E) and (H), connecting the PA to the descending aorta (DAo), while the Ao arch can be seen in (G) and (H). The blood pool volume shown in (A) is smaller than the true blood pool as segmentation was performed by signal intensity thresholding, which involved a trade‐off between cleanly separating blood in unconnected anatomy and faithfully tracing the outer extent of the blood pool. All boxes bounding the re‐sliced views measure 65 × 65 mm. The fetal heart is shown in radiological orientation, i.e., image axes up and right relative to the page correspond to left, anterior and/or superior anatomical directions. Views are shown using spatial B‐spline interpolation to avoid voxel distortion

Reconstructed 4D cines could be viewed in any arbitrary plane and at any phase in the cardiac cycle to examine cardiovascular morphology and connectivity. An example is shown in Figure 4 where the volume is viewed in variety of double‐oblique cardiac planes. The relative size of the chambers of the heart can be seen in horizontal long axis (4‐chamber view) and mid‐short axis views, with dilation and contraction of the left (LV) and right (RV) ventricles and left (LA) and right (RA) atria as the heart beats. Outflow tract and other off‐axis views reveal arterial and venous connections of the aorta (Ao), pulmonary artery (PA) and the superior (SVC) and inferior (IVC) vena cava, as well as the positions of the Ao and ductal arch (DA). The Supporting Information includes a video of the reconstructed 4D cine (Supporting Information Video S1) and intermediate results for cardiac synchronization, motion‐correction and outlier rejection (Supporting Information Figures S3‐S6) for this case.

**Figure 5:**
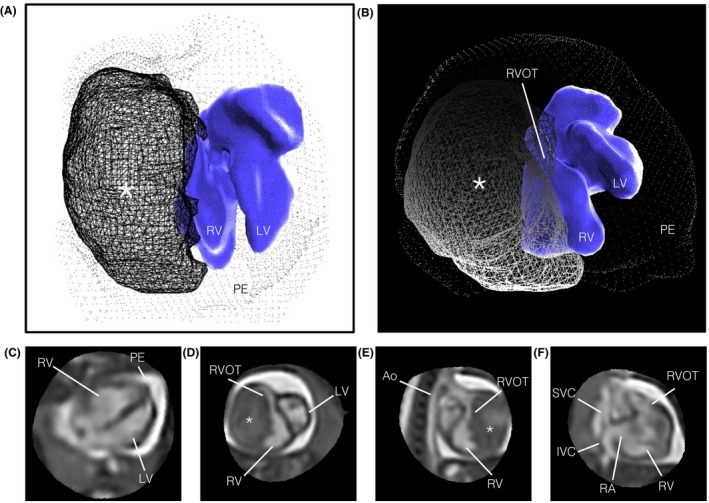
Reconstructed 4D cine of the heart of a $33^{+3}$ week gestational age fetus (ID11) with large fibroma, measuring 24 × 24 × 37 mm, involving the lateral wall of the right ventricle. A volume surface rendering is shown in (A) superior and (B) anterior views, for reference of relative shape and position of the cardiac blood pool (blue surface), fibroma (lined mesh) and pericardial effusion (dotted mesh). The reconstructed 4D cine is shown in (C) 4‐chamber, (D) short axis, (E) right ventricular outflow tract and (F) right 3‐chamber views. Extensive associated pericardial effusion (PE) can be seen as bright signal surrounding the heart. The fibroma (asterisk) is encapsulated within myocardial tissue and confined to the margin of the right ventricular (RV) cavity, causing external compression of the right side of the heart, including the RV, right atrium (RA) and right ventricular outflow tract (RVOT), while the left ventricle (LV), aorta (Ao), and both the superior (SVC) and inferior (IVC) vena cava appear normal. The blood pool volume rendering in (A) and (B) may be smaller than the true blood pool. All boxes bounding the re‐sliced views in (C‐F) measure 80 × 80 mm and the fetal heart are shown in radiological orientation, i.e., image axes towards the top and right of the page correspond to left, anterior and superior anatomical directions. Views are shown using spatial B‐spline interpolation to avoid voxel distortions

Figure 5 shows a fetus with a cardiac tumor (ID11). The ability to visualize the relationship of the mass and the myocardium in both space and time allowed for qualitative assessment of the impact of the tumour on cardiac function. A particularly large outlier class was estimated in this case (44% of frames rejected) compared to all other cases in cohort 1 (median 10% rejected, range 5‐28%); however this particular heart was grossly different in shape and appearance compared to the other fetal cases. It may be that the current implementation of outlier rejection is better suited to fine, high‐contrast anatomy, such as the normal fetal brain and heart, than to anatomy containing large regions with homogeneous signal.

**Figure 6:**
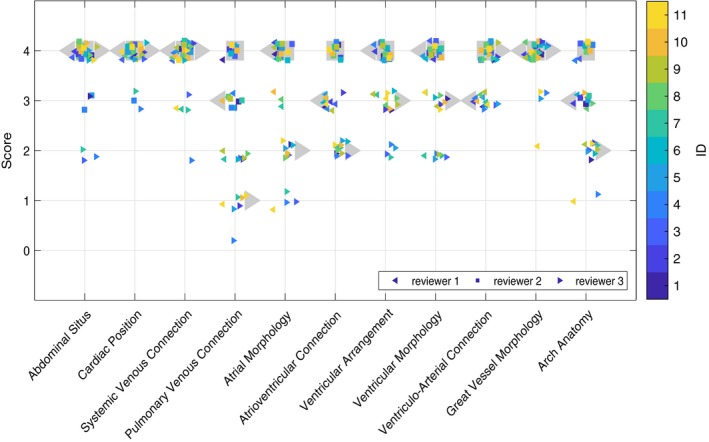
Reader score by evaluation category for cardiac 4D cines of 11 fetuses in cohort 1. Scores are shown for reader 1 (left‐pointing triangles), reader 2 (squares), and reader 3 (right‐pointing triangles), with 4, 7, and 1 years experience reading cardiac MRI, respectively. Median reader scores are shown as large grey markers while colored markers denote scores for individual fetal cases

The scoring evaluation is summarized in Figure 6 for fetal cases in cohort 1. Scores were generally high in all categories suggesting a potential to assess a range of cardiac anatomy in the 4D cines. Mean scores were between 3 and 4 for all fetal cases (median 3.4) in cohort 1 (Table 2) indicating that 4D cine MRI data were of adequate quality to determine most anatomical details. The lowest mean scores across all readers were for pulmonary venous connections, atrioventricular connections, and arch anatomy, suggesting that small anatomical features were the most challenging for the readers. The reconstructed 4D cines of the second cohort of fetal subjects were visually assessed by one reader (DL) and determined to be of similar high quality as the first cohort in the majority of cases, while insufficient data resulted in reduced quality in 2 cases for which 1920 (ID16) and 2590 (ID18) image frames were acquired covering the fetal heart, compared to a minimum of 3072 frames for all cases in cohort 1.

All steps of the proposed method were performed as described, with the exception of 2 cases in cohort 1 that required some motion‐corrupted data to be manually excluded prior to reconstruction (ID04,ID07). Prior to excluding motion‐corrupted slices these 2 cases had dev(**A**) = 3.8 mm (ID04) and 2.5 mm (ID07), larger than than all other cases in cohort 1 (median 1.3 mm, range 1.0‐2.3 mm). Manual intervention resulted in dev(**A**) = 1.8 mm (ID04) and 2.3 mm (ID07), and yielded visually improved 4D cine reconstructions in both cases, though both received low mean reader scores and had large outlier classes, as expected. Results from motion‐corrupted data are shown in Supporting Information Figure S7.

**Figure 7:**
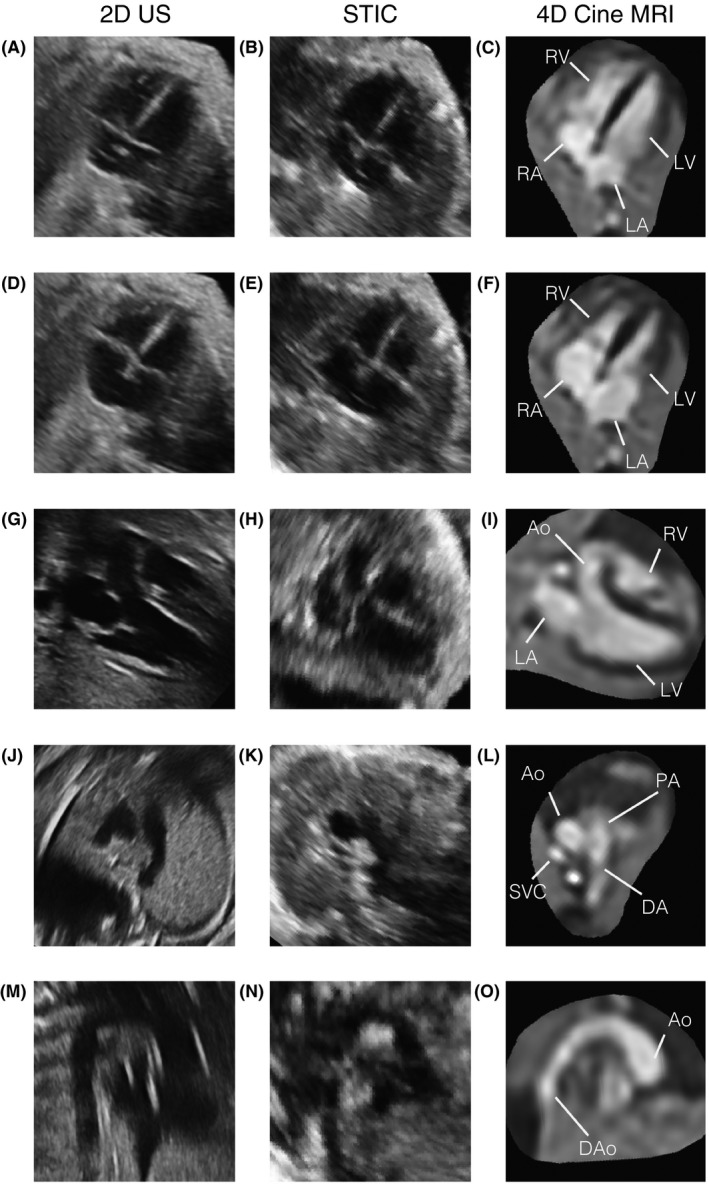
Comparison of MRI and ultrasound (US) in a $32^{+1}$ week gestational age fetus (ID01) with moderate dilation of the ascending aortic root and rightward tortuosity of the proximal ascending aorta. The fetal heart is shown in matched views in 2D echocardiogram (left column), 4D spatio‐temporal image correlation (STIC) ultrasound (centre column) and reconstructed 4D cine MRI (right column). Four‐chamber views at (A)‐(C) end‐ventricular diastole and (D)‐(F) end‐ventricular systole show the alternating contraction and expansion of the ventricles (LV, RV) and atria (LA, RA), with balanced chambers. (G)‐(I) Left 3‐chamber view, (J)‐(L) 3‐vessel view and (M)‐(O) aortic arch in sagittal view revealing a dilated aorta (Ao) at the sinotubular junction, compared to the pulmonary artery (PA), ductal arch (DA) and descending aorta (DAo). Some artefacts can be seen in the 2D US and STIC data, occasionally obscuring cardiac anatomy, while MRI had good signal coverage of the entire cardiovascular system. All bounding boxes measuring 65 × 65 mm and the fetal heart is shown in radiological orientation. The 4D MRI views are shown using spatial B‐spline interpolation to avoid voxel distortions

Reconstructed 4D cine MRI was compared with 2D and STIC ultrasound in the cases where matched data was acquired. An example is shown in Figure 7 where the three imaging methods are compared in matched views. Though STIC quality has been reported to be reduced in fetuses in the third trimester,^28^ the STIC volume of this $32^{+1}$ week gestational age fetus was the best quality of those acquired. In the other 6 fetal cases with matched ultrasound data, 2D echocardiography images were of comparable quality to those shown. However STIC data in 4 cases were clearly of poor quality, particularly in the through‐plane direction, presumably due to fetal motion, while MRI 4D reconstructions were of high quality in all 7 cases with matched US data.

Both 2D US and STIC showed clear definition of vessel and chamber boundaries, including valves, with high spatial and temporal resolution. Reconstructed cine MRI had good contrast between blood and surrounding tissue. However, visualization of valves and other small and rapidly moving anatomy was limited due to the spatio‐temporal resolution of the acquisition. While the real‐time aspect of 2D US made it robust to fetal motion, interpretation of anatomy was limited to the views acquired at the time of examination. By contrast, both STIC and 4D cine MRI allowed for offline analysis in arbitrary planes with full coverage of the heart and great vessels, facilitating understanding of the spatial relationships between cardiovascular structures.

Analyses of cardiac dimensions measured on US and MR are shown in Supporting Information Figure S8. There was good agreement between 4D MR and 2D US measurements for both readers (*p* > 0.10), with relatively small difference (bias < 5% of the mean distance) but large 95% limits of agreement (LOA ≈ 5 mm, or 35% of the mean distance), comparable to those observed in other studies of fetal MR and US.^8^ STIC data was not measured due to the poor quality in 4 of 7 cases. There was only moderate agreement between readers for both 2D US (*p* < 0.01, bias = −1.1 mm, LOA = 3.3 mm) and 4D MR (*p* ≈ 0.05, bias = −0.3 mm, LOA = 2.0 mm), which may also contribute to the large limits of agreement observed in the comparison of 4D MRI and 2D US. The variability of measurements on 2D US made by the two readers is in line with large inter‐observer errors in linear measurements in repeatably studies of fetal echocardiography,^29^ but may also be due to differences between diastolic/systolic frames selected. The variability of measurements made on 4D MRI may relate to differences in the plane used for the 4‐chamber view by the two readers.

**Figure 8:**
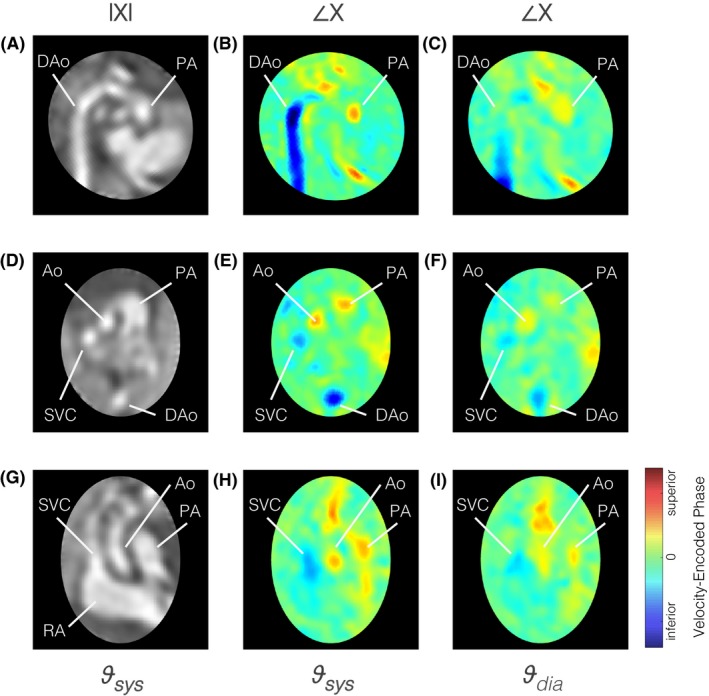
Pseudo 4D velocity mapping in a healthy $30^{+3}$ week gestational age fetus (ID02). Complex‐valued cine volume magnitude, |*X*|, and velocity‐encoded phase, ∠*X*, are shown in (A)‐(C) sagittal aortic arch plane, (D)‐(F) 3‐vessel view, and (G)‐(I) coronal plane perpendicular to 3‐vessel view. The phase of the reconstructed 4D cine was most sensitive to velocities in the fetal superior‐inferior direction, perpendicular to the target stack acquired transverse to the fetal trunk. Flow in the inferior direction (blue), is seen in the descending aorta (DAo) and superior vena cava (SVC), while flow in the superior direction (red) is seen in the ascending aorta (Ao) and pulmonary artery (PA). Peak flows can be seen in systole, $\upsilon_{\mathrm{sys}}$, with reduced flow during diastole, $\upsilon_{\mathrm{dia}}$. The right atrium (RA) is labelled in (G) for reference. The fetal heart is oriented in radiological convention with double‐oblique planes shown using spatial B‐spline interpolation to avoid voxel distortion

A complex‐valued 4D cine is shown in Figure 8 with the velocity‐encoded phase most sensitive to flow in the fetal superior‐inferior direction and the magnitude‐valued 4D cine shown as an anatomical reference. High flow can be seen in the great vessels in systole, with reduced flow in diastole.

## 4 DISCUSSION

Whole‐heart 4D reconstruction of the fetal heart from multi‐planar real‐time MRI was successful using the proposed framework in all fetal cases with at least 3000 image frames covering the heart. These reconstructed 4D cines could be visualized in any 2D plane without the need for highly‐specific scan plane prescription prior to acquisition or maternal breath hold to minimize motion. The three‐dimensional spatial aspect of these 4D cines allowed for interpretation of the connections of the cardiac chambers and vessels, while the temporal aspect improved the depiction of pulsatile anatomy.

Reconstructions of simulated MR images confirmed that spatial and temporal features could be reliably recovered, within the limits of the resolution of the acquisition. There was some blurring in the 4D cine data reconstructed from simulated MR images compared to the high‐resolution ground truth, which can be attributed to the low spatio‐temporal resolution sampling of the real‐time data. There was also a ringing‐like signal intensity effect in the 4D cine data that was apparent in the simulation results (Figure 3D), but had a more subtle impact on the fetal results. This effect may arise from the approximated in‐plane spatial PSF, however testing is not straightforward due to the complexity of implementing and computing a sinc PSF.

Overall high scores were assigned by three readers in an evaluation based on the segmental approach to defining cardiac anatomy and pathology. The lowest scores were assigned in categories focused on small anatomical features, such as pulmonary venous connections and arch anatomy, where spatio‐temporal resolution limited the depiction of fine details, as was also the case for small septal defects and all atrioventricular and ventriculo‐arterial valves. Though a category such as ventricular morphology received high scores in this evaluation, as there was enough other information to give the readers high confidence, particular abnormalities, such as valvular defects, were more visible in ultrasound images. However, some cardiac anatomy was obscured by artefact in the ultrasound images due to acoustic shadowing and, in the case of STIC data, blurring and misalignment. There was good agreement between two‐point measurements of ventricular lengths and widths on 2D M‐mode US and reconstructed 4D cine MRI, with little bias but large limits of agreement, and moderate inter‐observer variability for both modalities. However, the accuracy of MR measurements may be limited by spatio‐temporal resolution.

The results of the evaluation suggest that there is potential utility of the method to generate 4D cines that can be used for a comprehensive assessment of the fetal heart, either as an adjunct to ultrasound or in combination with other MRI techniques. For example, while aortic and ductal arch branching patterns may be difficult to determine in the 4D reconstruction, a static volume reconstruction of $T_{2}$‐weighted spin echo MRI^30^ may more clearly depict the details of the arch anatomy. However, 4D reconstruction captures cardiac movement across the entire cardiac cycle, which may improve visualization of anatomy affected by cardiac motion. In the future, the use of higher acquisition under‐sampling factors could be explored, possibly in combination with the use of flexible receive coil arrays,^31^ to determine how well the reconstruction copes with increased x‐f aliasing. These improvements will allow for higher resolution and strengthen the role of MRI in the assessment of the fetal heart in utero.

The proposed framework is fully automated aside from anatomical ROIs and identification of a target for initial stack‐stack registration. While these user‐interactions are not particularly laborious, they could be replaced with automated techniques for segmentation^32^, ^33^ and target stack identification using a measure of slice alignment.^34^ Manual intervention was required to exclude data with significant motion in 2 cases in cohort 1. In the original 4D cines reconstructed using these motion‐corrupted data, the anatomy was blurred and inconsistent as errors in stack‐ and slice‐wise motion‐correction lead to slice‐slice cardiac synchronization errors, and the reconstruction did not converge to a good depiction of the underlying fetal heart. A motion metric^34^ could be used in a preprocessing stage to identify slices with significant motion prior to any motion correction or cardiac synchronization without need for intervention. The focus of this work was on volumetric depiction of the whole fetal heart, and, consequently, the acquisition and reconstruction of k‐t SENSE MRI was not fundamentally changed from the 2D implementation^4^ and many of the potential improvements previously suggested still apply.

The use of a constant heart rate for each slice does not accommodate beat‐to‐beat variation resulting in small timing errors.^4^ Cardiac monitoring using doppler ultrasound gating^35^ or image‐based self‐gating^33^, ^36^, ^37^ could reduce these timing errors, either in combination with or instead of the current approach.

Simulation experiments showed slice‐slice cardiac synchronization error of less than 5% of the cardiac cycle for the range of displacements measured in the majority of fetal cases (Figure S2D), suggesting the proposed method provided reliable results. Cardiac synchronization was performed prior to volumetric reconstruction in the proposed framework and synchronization errors may be improved if the two steps were interleaved in a similar manner to frame‐volume registration.

Outlier rejection was performed as implemented by Kuklisova et al,^5^ with robust statistics shown to effectively reduce the influence of inconsistent data. As magnitude‐valued images were used, sensitivity to image artefacts that manifest in the phase of complex‐valued images was reduced compared to the complex‐valued outlier rejection used in the 2D framework.^4^ However, this did not appear to have a dramatic effect as real‐time images were reconstructed using spatially uniform k‐t SENSE regularization, thereby suppressing image artefact to some extent. Complex‐valued outlier rejection should be possible in a velocity‐sensitive reconstruction framework.

## 5 CONCLUSION

In summary, four‐dimensional representation of the fetal heart and great vessels was achieved using a highly‐accelerated multi‐planar real‐time MRI acquisition combined with retrospective motion correction, cardiac synchronization, outlier rejection and volumetric cine reconstruction in the image domain. The motion‐tolerant framework did not require maternal breath‐hold or precise scan planning during acquisition, and reconstruction was fully automated aside from user‐specified fetal heart and chest ROIs. The framework proved to be robust when used on fetal data and successfully generated good quality 4D cines in all fetal cases where sufficient data was collected covering the entire fetal heart. Readers had an overall high confidence in a comprehensive evaluation of the fetal heart using reconstructed 4D cine MRI and there was good agreement, but large limits of agreement, between two‐point measurements of ventricular dimensions on 2D M‐mode ultrasound and reconstructed 4D cine MRI.

Reconstructions from simulated MR images confirmed that correct spatial and temporal features could be reliably recovered, though there was some blurring due to the spatio‐temporal resolution of the acquired real‐time images. The use of image domain motion correction methods conferred significant advantages in terms of outlier rejection and the ability to accommodate quite large fetal motion, but had the downside of presenting major challenges for increasing the spatial and temporal resolution of the real‐time MR acquisition.

The proposed methods show promise as a framework for motion‐corrected reconstruction and 4D assessment of the fetal heart and great vessels. Promising results from a preliminary assessment of velocity‐sensitive volume reconstruction suggest potential for simultaneous reconstruction of a four‐dimensional cine and fully‐encoded velocity information from multi‐planar real‐time bSSFP MRI.

## Supporting information


**FIGURE S1** Normalized root mean square error (*NRMSE*) between 4D cine, $X^{*}$, reconstructed from simulated MR images using known transformations and cardiac phases, and the ground truth data, ***χ***. Error contour maps are shown for $X^{*[n_{\mathrm{SR}}]}$ after $n_{\mathrm{SR}}\;=\;5$ to 30 super‐resolution iterations for a range of values for regularization controlling parameter, $\tilde{\lambda }$, and edge definition parameter, *δ*, used for edge‐preserving regularization. Values of *δ* are given relative to mean signal intensity. The star indicates the selected reconstruction parameter values
**FIGURE S2** Assessment of 4D cine reconstruction using MR images simulated from numerical phantom. (A) Change in normalized root mean square error (*NRMSE*) between cine volume reconstructed using known transformations and cardiac phases, $X^{*[n_{\mathrm{SR}}]}$, and ground truth, $\chi^{*}$, with number of super‐resolution iterations, $n_{\mathrm{SR}}$. Each super‐resolution iteration took approximately 0.09 s per frame,for reconstructions performed with parallelization on an 8‐core CPU. Based on these results, ${\tilde{N}}_{\mathrm{SR}}\;=\;20$ iterations were used for the final volume reconstruction iteration when reconstructing fetal data. (B) *NRMSE* versus number of stacks used in reconstruction showing a small decrease in error with increasing number of stacks, suggesting that all stacks available be used in the reconstruction. (C) Root mean square error (*RMSE*) of estimated cardiac phases after slice‐slice cardiac synchronization versus mean displacement of $A^{*}$. The lowest errors occurred for $\text{disp}(A^{*})$ in the range of 2.3 to 9.3 mm with $RMSE\left(\theta ,\;\theta^{*}\right)\;<\;\frac{3}{32}\pi$, or approximately $0.05\;t^{\mathrm{RR}}$. The lowest $RMSE(\theta ,\;\theta^{*})$ was $\frac{1}{20}\pi$, for simulated MR images with $\text{disp}(A^{*})\;=\;5.6\;\text{mm}$, equivalent to 10 ms for the mean R‐R interval measured in the fetal study (404 ms). For reference, the median disp(**A**) measured in Cohort 1 was 5.8 ±1.8 mm. Very low levels of movement resulted in reduced overlap between slices and, consequently, misalignment of the cardiac cycle in slices that had very little overlap with all other slices. Conversely, the overlap between slices increased with some movement, resulting in an improvement in cardiac synchronization. However, large displacements lead to blurring in $X_{l}$ and an increase in the cardiac synchronization error for all slices. (D) The accuracy of motion correction was quantified as target registration error (*TRE*), defined as $TRE\left(A,\;A^{*}\right)\;=\;\sum_{j}\sum_{k}\text{dist}\left(A_{k}\left(y_{\mathrm{jk}}\right),\;A_{k}^{*}\left(y_{\mathrm{jk}}\right)\right)/\sum_{k}N_{j}$, where $\text{dist}\left(A_{k}\left(y_{\mathrm{jk}}\right),\;A_{k}^{*}\left(y_{\mathrm{jk}}\right)\right)$ is the spatial distance between the position of voxel $y_{\mathrm{jk}}$ transformed by $A_{k}$ and $A_{k}^{*}$. TRE is shown for estimated transformations for stack‐stack, slice‐volume, and frame‐volume registrations (blue line), with *NRMSE* between 4D cines reconstructed using estimated transformations and ground truth transformations using $N_{\mathrm{SR}}\;=\;20$ iterations (red line). TRE improved across the registration stages resulting in a final $TRE(A,\;A^{*})\;=\;1.34\;\text{mm}$, equivalent to 2/3 the acquired in‐plane resolution, after $N_{\mathrm{MC}}\;=\;3$ frame‐volume registration iterations, similar to the *TRE* measured previously for fetal brain volume reconstruction 0.72 mm for 1 mm in‐plane resolution images.${}^{5}$ A similar decrease in *NRMSE* was observed between 4D cine reconstructed with estimated transformations and ground truth transformations. No clear improvement was observed for higher number of motion correction iterations, suggesting that $N_{\mathrm{MC}}\;=\;3$ is sufficient
**FIGURE S3** Estimated heart rate (top row) for each slice acquired in a healthy $28^{+0}$ week gestational age fetus (ID09), with mean heart rate 150 ± 5 bpm (401 ± 13 ms). Unreliable heart rate estimates (red crosses) were identified using the height (middle row) and width (bottom row) of the peak in the temporal frequency spectrum used to estimate the heart rate, as shown in Figure 2. Threshold limits (horizontal lines, peak height 0.02, peak width 15.5 bpm) were calculated as three scaled median absolute deviations from the median. The heart rates for slices with peak heights less than the threshold were replaced with values linearly interpolated from temporally adjacent slices. Subsequently, heart rates for slices with peak widths above the threshold were replaced in a similar manner. Motion correction and outlier rejection results are shown in Figure S4 for the all image frames acquired in the time window indicated by the grey vertical band (38 to 83 seconds)
**FIGURE S4** Estimated transformations, displacements and image frame‐wise probabilities for six consecutive slices in one stack of multi‐planar real‐time MR images acquired in a $28^{+0}$ week gestational age fetus (ID09), corresponding to time window marked in Figure S3. An episode of fetal movement can be seen from acquisition time 72‐78 s in the fifth and sixth slices shown. Translations *tx*, *ty* and *tz* are with respect to scanner right‐left, anterior‐posterior and superior‐inferior directions, respectively. Rotations *rx*, *ry* and *rz* are about scanner y‐z, x‐z and x‐y axes, respectively. Translation and rotation of the average slice transformation are plotted as dotted lines. Displacement of image frame‐wise transformations, $\text{dev}\left(A_{k}\right)=\sum_{j}\mathrm{dist}\left(A_{l}\left(y_{\mathrm{jk}}\right),\;A_{k}\left(y_{\mathrm{jk}}\right)\right)/N_{j}$, and average slice transformations, $\text{disp}\left(A_{l}\right)=\sum_{k\in \text{slice}l}\sum_{j}\mathrm{dist}\left(A_{l}\left(y_{\mathrm{jk}}\right),\;A_{k}\left(y_{\mathrm{jk}}\right)\right)/\sum_{k\in \text{slice}l}N_{j}$, are plotted as solid and dotted lines, respectively. Deviation from the average slice transformation, $\text{dev}(A_{k})$, is shown relative to $\text{disp}(A_{l})$. In the slices shown, $\text{dev}(A_{l})\;=\;0.8$, 1.4, 0.6, 1.0, 2.8 and 1.6 mm. Arrows on the time axis of the fifth slice indicate the image frames shown on the top (a) and bottom Z (b) rows in Figure S5.
**FIGURE S5** Voxel‐wise error and probability maps for 2 image frames from the same slice acquired in high transverse plane in a $28^{+0}$ week gestational age fetus (ID09) at completion of 4D cine reconstruction. Image frames correspond to markers in Figure S4, for (A) frame acquired during a period with no fetal movement and (B) frame acquired during a period of fetal movement. Cropped views of the fetal heart show, from left to right, intensity‐corrected images ($Y_{k}^{*}$), images (${\hat{Y}}_{k}$) generated using Equation 1, error maps ($E_{k}$) and voxel‐wise probability maps ($P_{k}^{\mathrm{voxel}}$)
**FIGURE S6** Outlier rejection using voxel‐ and image frame‐wise robust statistics in a $28^{+0}$ week gestational age fetus (ID09) at completion of 3D cine reconstruction. (A) Voxel‐wise error distribution with likelihood, P(e) (solid green line), of observed error, *e*, modelled as the mixture of a Gaussian‐distributed inlier class (dashed green line) and uniformly distributed outlier class (dashed red line). Distribution parameters were estimated by maximizing the log‐likelihood of P(e) and used to map error to voxel‐wise probability, $p^{\mathrm{voxel}}$ (solid orange line). (B) Distribution of image frame potentials, *q*, with likelihood, P(q) (solid green line), modelled as the mixture of Gaussian‐distributed inlier (dashed green line) and outlier (dashed red line) classes, with expectation maximization of P(q) resulting in frame‐wise probability weighting, $p^{\mathrm{frame}}$ (solid orange line)
**FIGURE S7** Real‐time MR images and reconstructed 4D cine of a $29^{+6}$ week gestational age fetus (ID04) with isolated right aortic arch. Cropped views of selected image frames in one slice and line profile across the fetal heart and chest corresponding to yellow dashed line for all frames in the slice for (A) a slice with little fetal movement, $\text{dev}(A_{l})\;=\;0.9\;\text{mm}$, and (B) a slice with large fetal movements, $\text{dev}(A_{l})\;=\;10.1\;\text{mm}$, from the same stack. (C) Reconstruction using all acquired real‐time images resulting in a 4D cine corrupted by motion. (D) Exclusion of 19 of 54 total slices resulting in a 4D reconstruction with improved quality, particularly in the definition and detail of the arch anatomy in question. The heart is shown in (C) and (D) at end‐ventricular diastole in, from left to right: 4‐chamber view, short axis view, 3‐chamber view, high transverse view, similar to a 3‐vessel view, and as a line profile at the intersection of the long and short axes (dashed yellow lines) showing a cross section of the ventricles across cardiac phases (*ϑ*). The aortic (Ao) arch can be seen emerging from the left ventricle (LV), and then passing between the left atrium (LA) and right ventricle (RV) in the three vessel view. The vascular ring can be also be seen in the high transverse view, with the superior vena cava (SVC) at right and the duct arch (AD) passing to the left of the trachea (T), as normal, while the aorta (Ao) passes to the right. Manual exclusion of data was required in 2 of 20 cases (ID04,ID07)
**FIGURE S8** Comparison of cardiac dimensions measured on 2D M‐mode ultrasound (US) and reconstructed 4D cine MR. Left (LV) and right ventricular (RV) length (L) and diameter/width (W) were measured at end‐diastole (dia) and end‐systole (sys) by two readers. (A) Comparison of US and MR measures performed by reader 1. (B) Comparison of US and MR measures performed by reader 2. (C) Inter‐observer assessment for MR measures. (D) Inter‐observer assessment for US measures

**Video S1** Animation demonstrating spatial and temporal aspects of 4D whole‐heart cine reconstruction of healthy $28^{+0}$ week gestational age fetus (ID09) shown in Figure 4, with volume rendering of blood pool (red) for reference. The blood pool volume rendering may be smaller than the true blood pool
