# Supplementary material for: Fetal whole‐heart 4D imaging using motion‐corrected multi‐planar real‐time MRI
Source: Magn Reson Med. 2019 May 12;82(3):1055–72. doi: 10.1002/mrm.27798 (PMC6617816; doi:10.1002/mrm.27798)
Supplement: Supplementary file 1 — FIGURE S1 Normalized root mean square error (NRMSE) between 4D cine, X*, reconstructed from simulated MR images using known transformations and cardiac phases, and the ground truth data, χ. Error contour maps are shown for X*[nSR] after nSR=5 to 30 super‐resolution iterations for a range of values for regularization controlling parameter, λ~, and edge definition parameter, δ, used for edge‐preserving regularization. Values of δ are given relative to mean signal intensity. The star indicates the selected reconstruction parameter values FIGURE S2 Assessment of 4D cine reconstruction using MR images simulated from numerical phantom. (A) Change in normalized root mean square error (NRMSE) between cine volume reconstructed using known transformations and cardiac phases, X*[nSR], and ground truth, χ*, with number of super‐resolution iterations, nSR. Each super‐resolution iteration took approximately 0.09 s per frame,for reconstructions performed with parallelization on an 8‐core CPU. Based on these results, N~SR=20 iterations were used for the final volume reconstruction iteration when reconstructing fetal data. (B) NRMSE versus number of stacks used in reconstruction showing a small decrease in error with increasing number of stacks, suggesting that all stacks available be used in the reconstruction. (C) Root mean square error (RMSE) of estimated cardiac phases after slice‐slice cardiac synchronization versus mean displacement of A*. The lowest errors occurred for disp(A*) in the range of 2.3 to 9.3 mm with RMSE(θ,θ*)<332π, or approximately 0.05tRR. The lowest RMSE(θ,θ*) was 120π, for simulated MR images with disp(A*)=5.6mm, equivalent to 10 ms for the mean R‐R interval measured in the fetal study (404 ms). For reference, the median disp(A) measured in Cohort 1 was 5.8 ±1.8 mm. Very low levels of movement resulted in reduced overlap between slices and, consequently, misalignment of the cardiac cycle in slices that had very little overlap with all other slices. Conversely [file MRM-82-1055-s001.pdf]

SUPPORTING INFORMATION

Video S1. Animation demonstrating spatial and temporal aspects of 4D whole-heart cine reconstruction of healthy 28^{+0} week gestational age fetus (ID09) shown in Figure 4, with volume rendering of blood pool (red) for reference. The blood pool volume rendering may be smaller than the true blood pool.

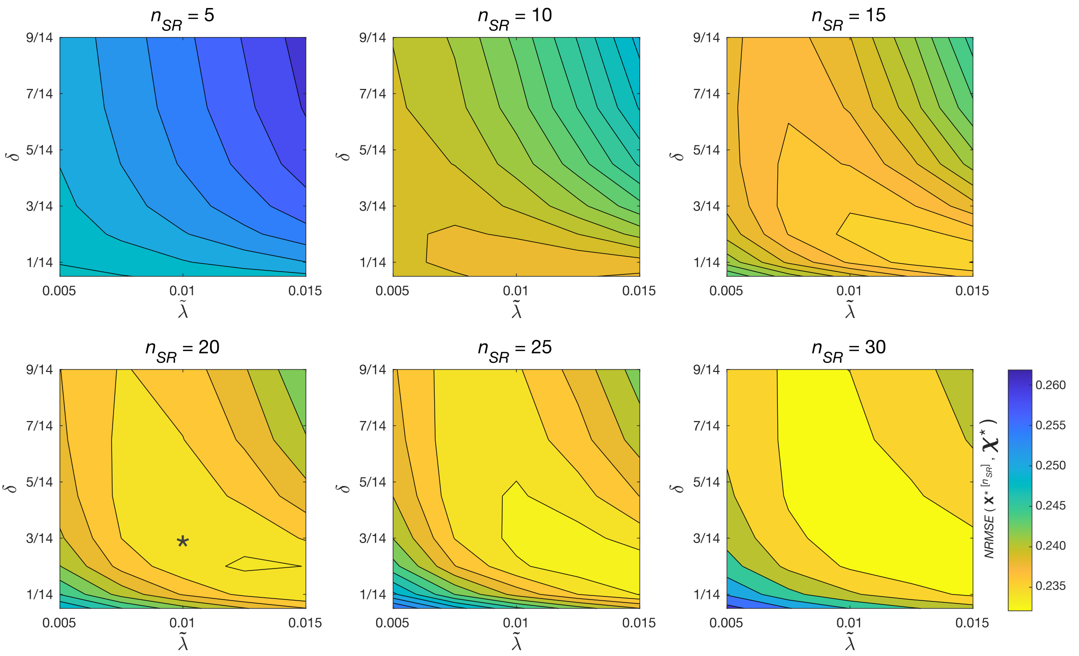

FIGURE S1 Normalised root mean square error ($NRMSE$) between 4D cine, $\mathbf{X}^*$, reconstructed from simulated MR images using known transformations and cardiac phases, and the ground truth data, χ. Error contour maps are shown for $\mathbf{X}^{*\,[n_{SR}]}$ after n_{SR} = 5 to 30 super-resolution iterations for a range of values for regularisation controlling parameter, $\tilde{\lambda}$, and edge definition parameter, δ, used for edge-preserving regularisation. Values of δ are given relative to mean signal intensity. The star indicates the selected reconstruction parameter values.

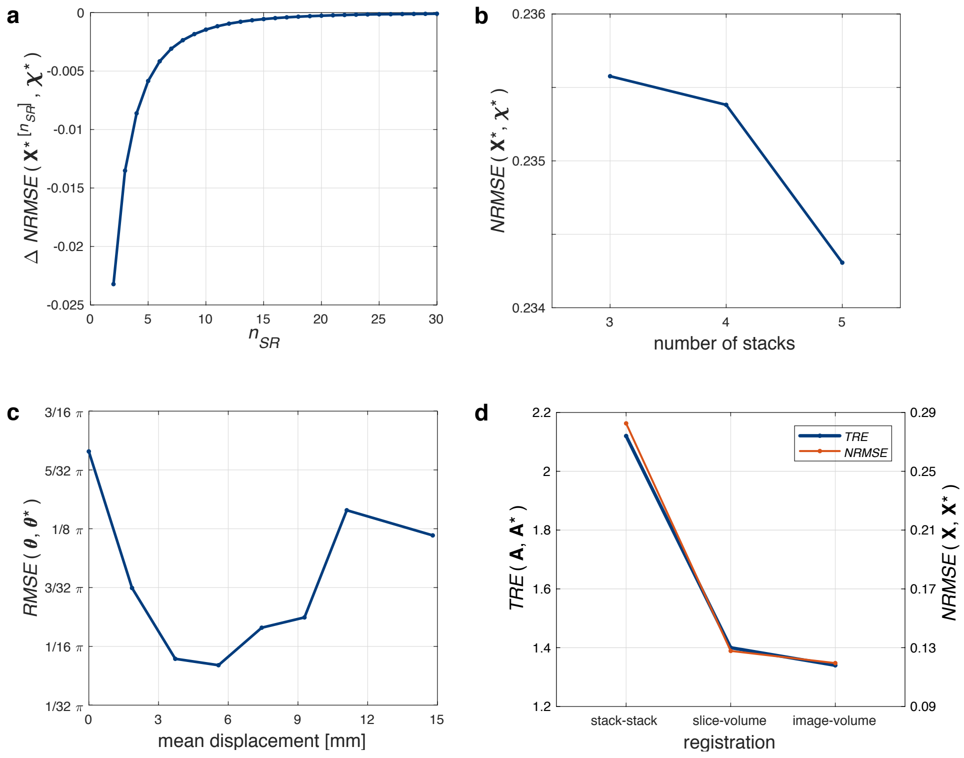

FIGURE S2 Assessment of 4D cine reconstruction using MR images simulated from numerical phantom. (**a**) Change in normalised root mean square error ($NRMSE$) between cine volume reconstructed using known transformations and cardiac phases, $\mathbf{X}^{*\,[n_{\mathrm{SR}}]}$, and ground truth, χ^*, with number of super-resolution iterations, n_{SR}. Each super-resolution iteration took approximately 0.09 s per frame,for reconstructions performed with parallelisation on an 8-core CPU. Based on these results, $\widetilde{N}_{\mathrm{SR}}$ = 20 iterations were used for the final volume reconstruction iteration when reconstructing fetal data. (**b**) $NRMSE$ versus number of stacks used in reconstruction showing a small decrease in error with increasing number of stacks, suggesting that all stacks available be used in the reconstruction. (**c**) Root mean square error ($RMSE$) of estimated cardiac phases after slice-slice cardiac synchronisation versus mean displacement of $\mathbf{A}^*$. The lowest errors occurred for disp($\mathbf{A}^*$) in the range of 2.3 to 9.3 mm with $RMSE(\boldsymbol{\theta}, \boldsymbol{\theta}^*) < \frac{3}{32}\pi$, or approximately 0.05 t^{RR}. The lowest $RMSE(\boldsymbol{\theta}, \boldsymbol{\theta}^*)$ was $\frac{1}{20}\pi$, for simulated MR images with disp($\mathbf{A}^*$) = 5.6 mm,, equivalent to 10 ms for the mean R-R interval measured in the fetal study (404 ms). For reference, the median disp($\mathbf{A}$) measured in Cohort 1 was 5.8 ± 1.8 mm. Very low levels of movement resulted in reduced overlap between slices and, consequently, misalignment of the cardiac cycle in slices that had very little overlap with all other slices. Conversely, the overlap between slices increased with some movement, resulting in an improvement in cardiac synchronisation. However, large displacements lead to blurring in $\mathbf{X}_l$ and an increase in the cardiac synchronisation error for all slices. (**d**) The accuracy of motion correction was quantified as target registration error (TRE), defined as $TRE\,(\mathbf{A}, \mathbf{A}^*) = \sum_j \sum_k \mathrm{dist}\left(\mathrm{A}_k(y_{jk}), \mathrm{A}^*_k(y_{jk})\right) / \sum_k N_j$, where dist $\left(\mathrm{A}_k(y_{jk}), \mathrm{A}^*_k(y_{jk})\right)$ is the spatial distance between the position of voxel y_{jk} transformed by A_k and A^*_k. TRE is shown for estimated transformations for stack-stack, slice-volume, and frame-volume registrations (blue line), with $NRMSE$ between 4D cines reconstructed using estimated transformations and ground truth transformations using N_{SR} = 20 iterations (red line). TRE improved across the registration stages resulting in a final $TRE(\mathbf{A}, \mathbf{A}^*)$ = 1.34 mm, equivalent to 2/3 the acquired in-plane resolution, after N_{MC} = 3 frame-volume registration iterations, similar to the TRE measured previously for fetal brain volume reconstruction 0.72 mm for 1 mm in-plane resolution images [5]. A similar decrease in $NRMSE$ was observed between 4D cine reconstructed with estimated transformations and ground truth transformations. No clear improvement was observed for higher number of motion correction iterations, suggesting that N_{MC} = 3 is sufficient.

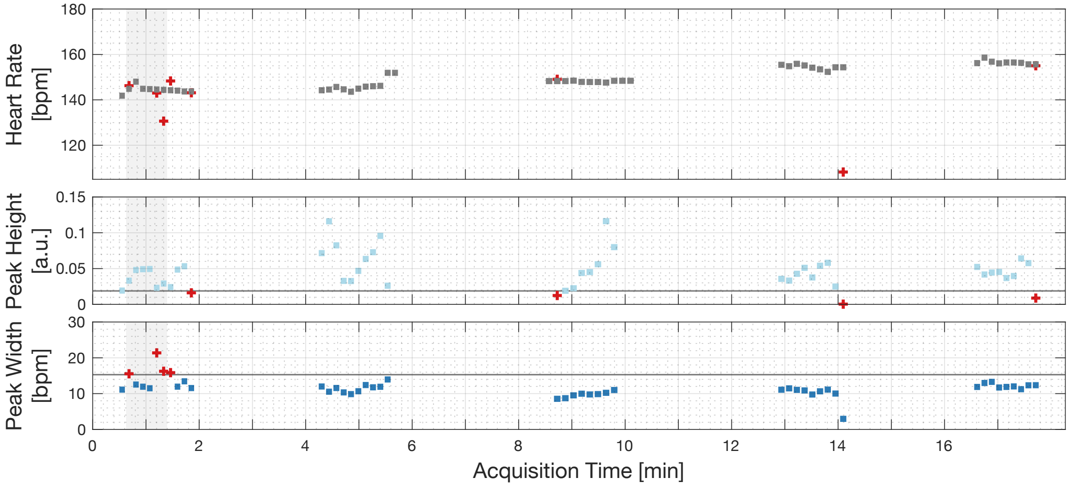

FIGURE S3 Estimated heart rate (top row) for each slice acquired in a healthy 28^{+0} week gestational age fetus (ID09), with mean heart rate 150±5 bpm (401±13 ms). Unreliable heart rate estimates (red crosses) were identified using the height (middle row) and width (bottom row) of the peak in the temporal frequency spectrum used to estimate the heart rate, as shown in Fig. 2. Threshold limits (horizontal lines, peak height 0.02, peak width 15.5 bpm) were calculated as three scaled median absolute deviations from the median. The heart rates for slices with peak heights less than the threshold were replaced with values linearly interpolated from temporally adjacent slices. Subsequently, heart rates for slices with peak widths above the threshold were replaced in a similar manner. Motion correction and outlier rejection results are shown in Fig. S4 for the all image frames acquired in the time window indicated by the grey vertical band (38 to 83 seconds).

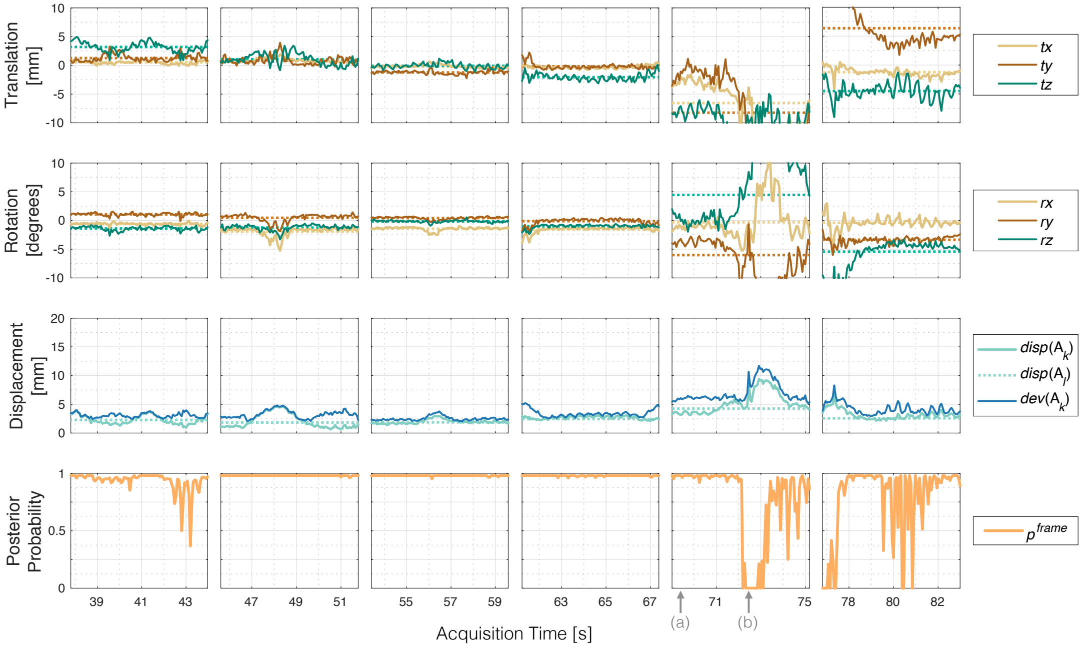

FIGURE S4 Estimated transformations, displacements and image frame-wise probabilities for six consecutive slices in one stack of multi-planar real-time MR images acquired in a 28^{+0} week gestational age fetus (ID09), corresponding to time window marked in Fig. S3. An episode of fetal movement can be seen from acquisition time 72-78 s in the fifth and sixth slices shown. Translations *tx*, *ty* and *tz* are with respect to scanner right-left, anterior-posterior and superior-inferior directions, respectively. Rotations *rx*, *ry* and *rz* are about scanner y-z, x-z and x-y axes, respectively. Translation and rotation of the average slice transformation are plotted as dotted lines. Displacement of image frame-wise transformations, $dev(\mathrm{A}_k) = \sum_j dist\left(\mathrm{A}_l(y_{jk}), \mathrm{A}_k(y_{jk})\right) / N_j$, and average slice transformations, $\mathrm{disp}(\mathrm{A}_l) = \sum_{k \in \mathrm{slice}\,l} \sum_j dist\left(\mathrm{A}_l(y_{jk}), \mathrm{A}_k(y_{jk})\right) / \sum_{k \in \mathrm{slice}\,l} N_j$, are plotted as solid and dotted lines, respectively. Deviation from the average slice transformation, $\mathrm{dev}(\mathrm{A}_k)$, is shown relative to $\mathrm{disp}(\mathrm{A}_l)$. In the slices shown, $\mathrm{dev}(\mathbf{A}_l)$ = 0.8, 1.4, 0.6, 1.0, 2.8 and 1.6 mm. Arrows on the time axis of the fifth slice indicate the image frames shown on the top (a) and bottomZ(b) rows in Fig. S5.

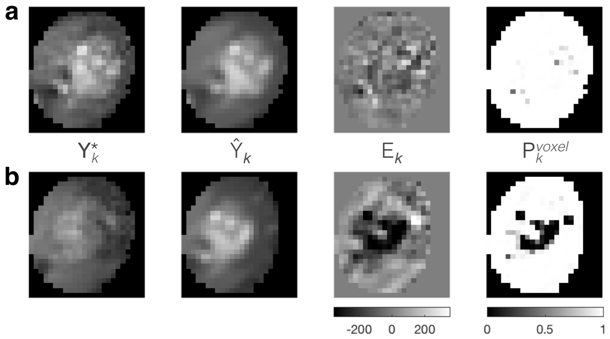

FIGURE S5 Voxel-wise error and probability maps for two image frames from the same slice acquired in high transverse plane in a 28^{+0} week gestational age fetus (ID09) at completion of 4D cine reconstruction. Image frames correspond to markers in Fig. S4, for (**a**) frame acquired during a period with no fetal movement and (**b**) frame acquired during a period of fetal movement. Cropped views of the fetal heart show, from left to right, intensity-corrected images (Y^*_k), images ($\widehat{Y}_k$) generated using Eq. 1, error maps (E_k) and voxel-wise probability maps (P^{voxel}_k).

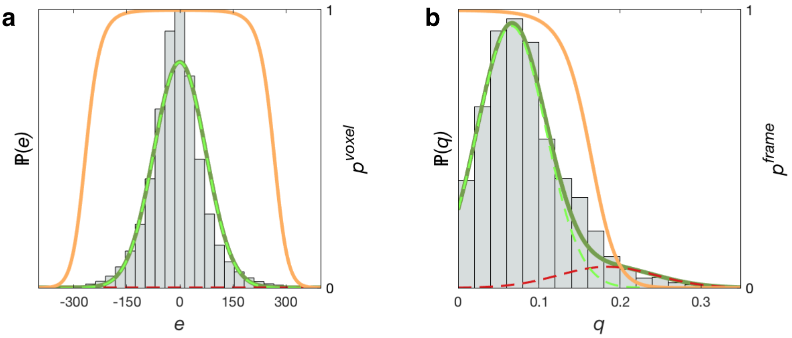

FIGURE S6 Outlier rejection using voxel- and image frame-wise robust statistics in a 28^{+0} week gestational age fetus (ID09) at completion of 3D cine reconstruction. (**a**) Voxel-wise error distribution with likelihood, $\mathbb{P}(e)$ (solid green line), of observing error, e, modelled as the mixture of a Gaussian-distributed inlier class (dashed green line) and uniformly-distributed outlier class (dashed red line). Distribution parameters were estimated by maximising the log-likelihood of $\mathbb{P}(e)$ and used to map error to voxel-wise probability, p^{voxel} (solid orange line). (**b**) Distribution of image frame potentials, q, with likelihood, $\mathbb{P}(q)$ (solid green line), modelled as the mixture of Gaussian-distributed inlier (dashed green line) and outlier (dashed red line) classes, with expectation maximisation of $\mathbb{P}(q)$ resulting in frame-wise probability weighting, p^{frame} (solid orange line).

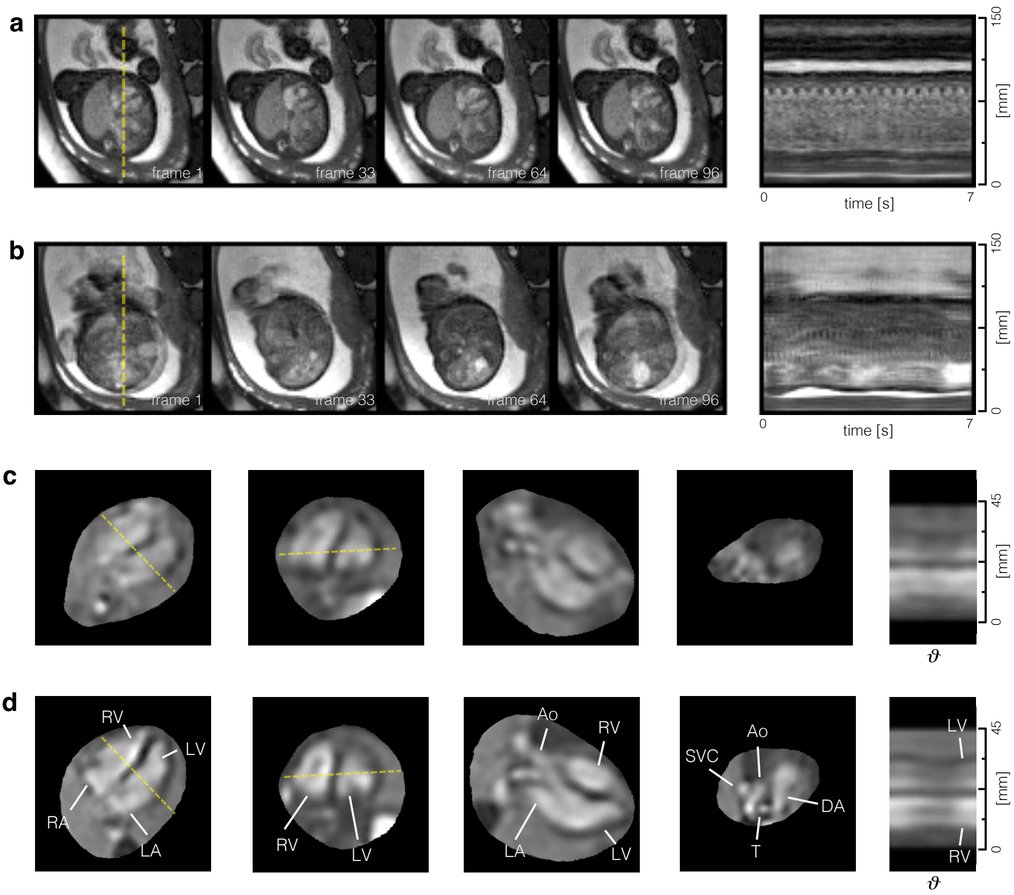

FIGURE S7 Real-time MR images and reconstructed 4D cine of a 29^{+6} week gestational age fetus (ID04) with isolated right aortic arch. Cropped views of selected image frames in one slice and line profile across the fetal heart and chest corresponding to yellow dashed line for all frames in the slice for (**a**) a slice with little fetal movement, dev($\mathbf{A}_l$) = 0.9 mm, and (**b**) a slice with large fetal movements, dev($\mathbf{A}_l$) = 10.1 mm, from the same stack. (**c**) Reconstruction using all acquired real-time images resulting in a 4D cine corrupted by motion. (**d**) Exclusion of 19 of 54 total slices resulting in a 4D reconstruction with improved quality, particularly in the definition and detail of the arch anatomy in question. The heart is shown in (c) and (d) at end-ventricular diastole in, from left to right: four chamber view, short axis view, three chamber view, high transverse view, similar to a three vessel view, and as a line profile at the intersection of the long and short axes (dashed yellow lines) showing a cross section of the ventricles across cardiac phases (ϑ). The aortic (Ao) arch can be seen emerging from the left ventricle (LV), and then passing between the left atrium (LA) and right ventricle (RV) in the three vessel view. The vascular ring can be also be seen in the high transverse view, with the superior vena cava (SVC) at right and the duct arch (AD) passing to the left of the trachea (T), as normal, while the aorta (Ao) passes to the right. Manual exclusion of data was required in two of twenty cases (ID04,ID07).

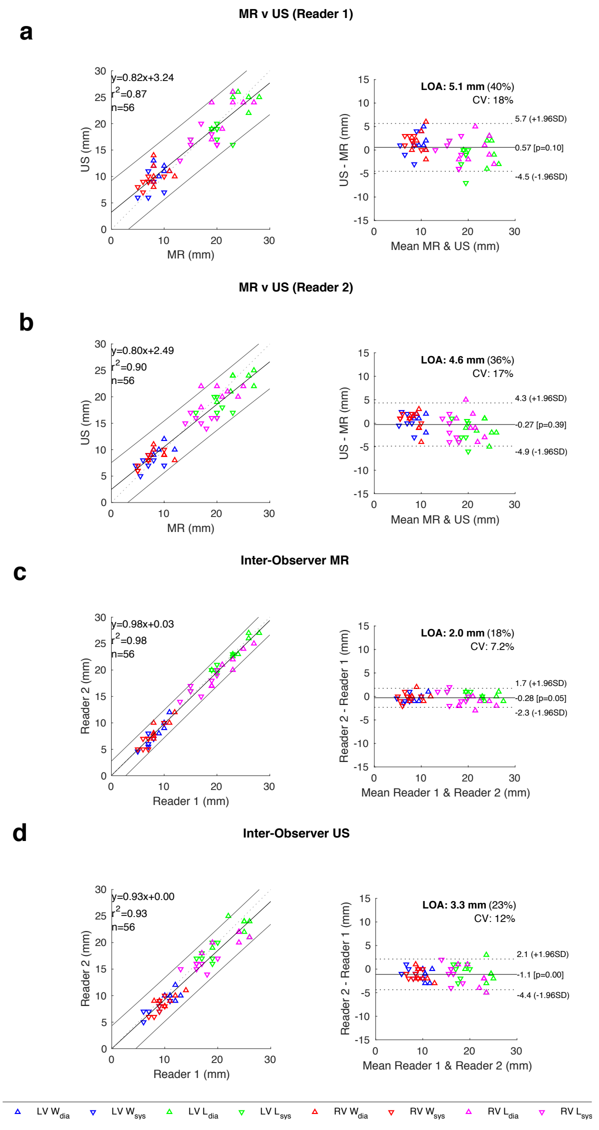

FIGURE S8 Comparison of cardiac dimensions measured on 2D M-mode ultrasound (US) and reconstructed 4D cine MR. Left (LV) and right ventricular (RV) length (L) and diameter/width (W) were measured at end-diastole (dia) and end-systole (sys) by two readers. (**a**) Comparison of US and MR measures performed by reader 1. (**b**) Comparison of US and MR measures performed by reader 2. (**c**) Inter-observer assessment for MR measures. (**d**) Inter-observer assessment for US measures.
